# Toxin release by conditional remodelling of ParDE1 from *Mycobacterium tuberculosis* leads to gyrase inhibition

**DOI:** 10.1093/nar/gkad1220

**Published:** 2023-12-19

**Authors:** Izaak N Beck, Tom J Arrowsmith, Matthew J Grobbelaar, Elizabeth H C Bromley, Jon Marles-Wright, Tim R Blower

**Affiliations:** Department of Biosciences, Durham University, South Road, Durham DH1 3LE, UK; Department of Biosciences, Durham University, South Road, Durham DH1 3LE, UK; Department of Biosciences, Durham University, South Road, Durham DH1 3LE, UK; Department of Physics, Durham University, Stockton Road, Durham DH1 3LE, UK; Biosciences Institute, Newcastle University, Framlington Place, Newcastle upon Tyne NE2 4HH, UK; Department of Biosciences, Durham University, South Road, Durham DH1 3LE, UK

## Abstract

*Mycobacterium tuberculosis*, the causative agent of tuberculosis, is a growing threat to global health, with recent efforts towards its eradication being reversed in the wake of the COVID-19 pandemic. Increasing resistance to gyrase-targeting second-line fluoroquinolone antibiotics indicates the necessity to develop both novel therapeutics and our understanding of *M. tuberculosis* growth during infection. ParDE toxin–antitoxin systems also target gyrase and are regulated in response to both host-associated and drug-induced stress during infection. Here, we present microbiological, biochemical, structural, and biophysical analyses exploring the ParDE1 and ParDE2 systems of *M. tuberculosis* H37Rv. The structures reveal conserved modes of toxin–antitoxin recognition, with complex-specific interactions. ParDE1 forms a novel heterohexameric ParDE complex, supported by antitoxin chains taking on two distinct folds. Curiously, ParDE1 exists in solution as a dynamic equilibrium between heterotetrameric and heterohexameric complexes. Conditional remodelling into higher order complexes can be thermally driven *in vitro*. Remodelling induces toxin release, tracked through concomitant inhibition and poisoning of gyrase activity. Our work aids our understanding of gyrase inhibition, allowing wider exploration of toxin–antitoxin systems as inspiration for potential therapeutic agents.

## Graphical Abstract

**Graphical Abstract:**
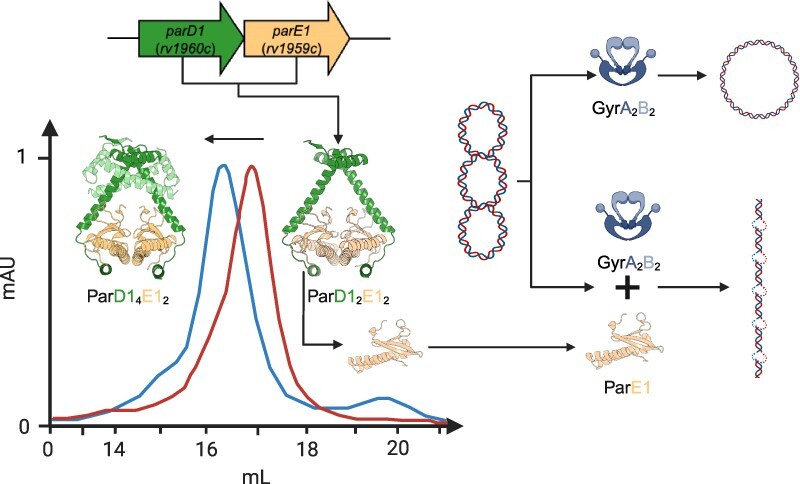

## Introduction

Despite concerted efforts, tuberculosis remains a major cause of morbidity and a leading cause of mortality worldwide (1). There were approximately 10.6 million new cases of tuberculosis in 2021, and it is estimated that over a quarter of the world's population would demonstrate an immunological response to the causative agent *Mycobacterium tuberculosis* (1). DNA gyrase is the single type II topoisomerase encoded by *M. tuberculosis* and remains an important drug target (2–4). DNA gyrase is responsible for the maintenance of DNA topology (5), and alters supercoiling through cycles of generating and re-ligating double-stranded (ds) breaks in DNA (6). This essential process that produces potentially cytotoxic dsDNA breaks has made type II topoisomerases an attractive target in both antimicrobial (7) and anti-cancer (8) drug research.

Toxin-antitoxin (TA) systems are found in most bacterial genomes and generally comprise small bicistronic loci encoding a protein toxin and a protein or RNA antitoxin (9–12). TA systems have key roles in native host bacterial growth, with their activity regulating a range of cellular processes (13–16). The specific physiological roles of these systems are often debated, and vary from organism to organism, but they have been associated with maintaining genomic stability, bacteriophage defence, biofilm formation, and bacterial persistence (17–21). TA systems have an apparent association with pathogenicity, especially in Mycobacteria, as *M. tuberculosis* H37Rv encodes an estimated 88 systems (22) (∼2% of the proteome), whereas the typically non-pathogenic *M. smegmatis* encodes an estimated 5 systems (∼0.08% of the proteome) (23,24).

The widespread and highly conserved ParDE TA systems, part of the RelE/ParE superfamily (25), target DNA gyrase (26,27). *M. tuberculosis* encodes two ParDE systems and several studies on the ParE1 and ParE2 toxins have shown their ability to inhibit gyrase enzymes from *E. coli*, *M. smegmatis* and *M. tuberculosis* (22,28–29). The *M. tuberculosis* ParDE systems have both been shown to be regulated in response to environmental stresses associated with infection (30–33). The *parE1* toxin gene was also identified through mutational studies as both important for survival in activated macrophages, and in dissemination to the spleen (33,34), potentially contributing to extrapulmonary tuberculosis. Expression profiling highlighted the ParE toxin genes, *parE1* and *parE2*, as some of the highest differentially regulated genes (second only to *rv1045* encoding toxin MenT3 (35)) when *M. tuberculosis* was subjected to starvation, acidification, and first-line drug exposure in combinations for varying time-lengths (30).

Here, we present microbiological, biochemical, structural and biophysical characterisation of the ParDE1 and ParDE2 TA systems from *M. tuberculosis*. Our studies show conserved modes of antitoxin recognition with distinct, complex-specific, interactions. We demonstrate a unique quaternary ParDE structure, with ParDE1 forming a heterohexameric complex. Unexpectedly, we were readily able to detect and characterise higher order structures for the ParDE1 complex. Conditional remodelling of the ParDE1 complex can be thermally induced, allowing control of the dynamics of complex formation, driving ParDE1 from a heterotetrameric state into higher order structures. By combining the remodelling process with gyrase assays we demonstrate selective ParE1 release. These results expand our understanding of *in vitro* toxin–antitoxin activity, and suggest potential routes for gyrase inhibition.

## Materials and methods

### Bacterial strains and culture conditions

*E. coli* strains DH5α (Invitrogen), Rosetta 2 (DE3) pLysS (Novagen), and ER2566 (New England Biolabs) were grown at 37°C, and *M. smegmatis* mc^2^155 (ATCC 700084) was grown at 37°C or 30°C, either on agar plates or shaking at 220 rpm. Lysogeny broth (LB) was used as the standard growth media for overnight liquid cultures, with 1.5% w/v agar added for solid agar plates. 2× YT was used for protein expression cultures. Growth was monitored using a spectrophotometer (WPA Biowave C08000) measuring optical density at 600 nm (OD_600_). When necessary, growth media was supplemented with ampicillin (Ap, 50 μg/ml), chloramphenicol (Cm, 25 μg/ml), streptomycin (Sm, 50 μg/ml), kanamycin (Km, 50 μg/ml), Tween-80 (0.2% v/v), isopropyl-β-d-thiogalactopyranoside (IPTG, 1 mM), or anhydrotetracycline (ATc, 100 ng/ml).

### DNA isolation and manipulation

Plasmid DNA was purified from transformed DH5α cells using a NEB Monarch® Plasmid MiniPrep kit following the manufacturer's instructions. Larger amounts of negatively supercoiled plasmid (pSG483) DNA for assays was purified from transformed DH5α cells using a Machery-Nagel NucleoBond Xtra Midi Plus EF kit following the manufacturer's instructions. Plasmids were eluted in dH_2_O for storage at −20°C. Plasmids pTRB316 and pTRB696 were made previously (3). Plasmids pTRB568, pTRB569 and pTRB570 were generated commercially at Genscript using sequences optimised for *E. coli* expression. Plasmid derivatives of pJEM15 and pGMC were also generated commercially by Genscript. Plasmids are described in Supplementary Table S1.

### β-galactosidase reporter assays

Co-transformants of *M. smegmatis* mc^2^155 containing either pJEM15 vector-only (36), or pJEM15-P*_rv1960c_*_/_*_rv1959c_* (1000 bp upstream of *parDE1*), together with pGMC vector-only (35), pGMC-*parD1*, pGMC-*parE1* or pGMC-*parDE1*, and *M. smegmatis* mc^2^155 containing either pJEM15 vector-only or pJEM15-P*_rv2142A_*_/_*_rv2142c_* (1000 bp upstream of *parDE2*), together with pGMC vector-only, pGMC-*parD2*, pGMC-*parE2* or pGMC-*parDE2*, were screened for β-galactosidase activity on LB-agar plates containing the relevant antibiotics, Tween-80 (0.05% v/v), isopropyl ß-d-1-thiogalactopyranoside (IPTG, 1 mM), and 5-bromo-4-chloro-3-indolyl β-d-galactopyranoside (X-Gal, 40 μg/ml) for blue/white qualitative visualisation of β-galactosidase activity, either in the absence or presence of anhydrotetracycline (ATc; 100 ng/ml). Colonies were used to inoculate LB media supplemented with 0.05% Tween-80 and 0.2% glycerol, then grown at 37°C with 180 rpm shaking until reaching saturation. Cultures were then diluted 1:50 v/v into fresh growth media and further incubated until OD_600_ = 0.8. Cells from 2 ml of each culture were collected by centrifugation (4200 × g, 10 min, 4°C), re-suspended in 2 ml ice-cold Z-buffer (6 mM NaH_2_PO_4_.H_2_O, 10 mM KCl, 50 mM β-mercaptoethanol, 1 mM MgSO_4_, pH 7.0), and mechanically lysed using a FastPrep-24 5G homogeniser (MP Biomedicals™). Cell lysate was decanted into fresh tubes, and the reaction started following the addition of 100 μl ortho-Nitrophenyl-β-galactoside (ONPG, 4 mg/ml). Tubes were incubated at 30°C for 30 min before termination following the addition of 200 μl 1 M Na_2_CO_3_. The reaction was then centrifuged at 13 000 × g for 5 min to remove cellular debris, with 500 μl of supernatant transferred into a clean cuvette and diluted with 500 μl Z-buffer for measurement of OD_420_ and OD_550_ values (DeNovix DS-11 + spectrophotometer) and subsequent calculation of activity as described (37).

### Preparation of nicked and linear form pSG483

For nicking, 10 μg pSG483 was incubated with 10 units of Nb.Bpu10I (ThermoFisher) in 1 x Buffer R (ThermoFisher) for 1 hr at 37°C. The enzyme was deactivated by a further incubation step at 80°C for 20 min. For linearisation, 10 μg pSG483 was incubated with 10 units of BamHI-HF® (NEB) in 1 x CutSmart buffer (NEB) for 1 h at 37°C. The enzyme was deactivated after incubation by a further incubation step at 65°C for 10 min. Conversion of supercoiled pSG483 into appropriate products was assessed by agarose gel electrophoresis. Both nicked and linear form pSG483 were subsequently stored at −20°C.

### Preparation of relaxed form pSG483

Initially, 50 μg pSG483 was nicked by incubation with 10 units Nb.Bpu10I (ThermoFisher) in 1× Buffer R (ThermoFisher) for 4 h at 37°C. The enzyme was deactivated by a further incubation step at 80°C for 20 min. The reaction was allowed to cool to room temperature before being supplemented with ATP to a final concentration of 1 mM. 10 μl T4 DNA ligase was added and the reaction was left at room temperature for 16 h. After ligation, ethanol precipitation was performed to remove proteins. An equal volume of UltraPure™ phenol:chloroform:isoamyl alcohol (25:24:1, vol/vol/vol) (ThermoFisher) was added to the reaction mixture before vortexing briefly. The sample was centrifuged at 16 000 × g for 2 min and the resulting aqueous layer was removed and carried forward. An equal volume of chloroform (ThermoFisher) was added to the aqueous layer before centrifugation at 16 000 × g for 2 min. The resulting aqueous layer was carried forward and 1/10 volume 3 M sodium acetate pH 5.2 was added. Then, 2 volumes of 100% ethanol were added, briefly mixed by pipetting, and stored at -80°C for 30 min. The sample was centrifuged at 16 000 × g and 4°C for 20 min. The ethanol was removed, and the DNA pellet dried at room temperature. The DNA pellet was resuspended in room temperature dH_2_O to approximately 300 ng/μl.

### Protein expression

Proteins were expressed and purified following published protocols (3), with small variations as appropriate. For the expression of the gyrase subunit proteins, GyrA and GyrB, Rosetta™ 2 pLysS cells were transformed with pTRB696 and pTRB316, respectively. Both gyrase subunits were expressed with a TEV protease cleavable N-terminal hexahistidine (6His) tag for purification. Cells were grown at 37°C with shaking at 180 rpm to an optical density (OD_600_) of 0.6 at which point the incubation temperature was reduced to 30°C and IPTG was added to a final concentration of 0.8 mM to induce overexpression. Cells were subsequently grown for a further 4 hr at 30°C with shaking at 160 rpm.

Each of the toxin–antitoxin systems in this study were expressed from Duet vectors (Supplementary Table S1). Rosetta™ 2 pLysS cells were transformed with the appropriate plasmids for the expression of the ParDE1 complex (pTRB569) or ParDE2 complex (pTRB570). Toxins ParE1 and ParE2 were expressed with a hSENP-2 cleavable N-terminal 6His-SUMO tag for purification of the complexes (35). Cells were grown at 37°C with shaking at 180 rpm to an optical density (OD_600_) of 0.6 at which point the incubation temperature was reduced to 18°C and IPTG was added to a final concentration of 1 mM to induce overexpression. Cells were subsequently grown for a further 16 h at 18°C with shaking at 160 rpm. To express the ParDE1 complex in the heterotetramer stoichiometry, the protocol above was followed with the following adjustments: IPTG was added to a final concentration of 0.5 mM and expression temperature was lowered to 16°C.

### Protein purification

Bacterial cells were pelleted from liquid culture by centrifugation at 4200 × g for 30 min at 4ºC. Cell pellets were resuspended in lysis buffer A500 [20 mM Tris base pH 8.0, 500 mM NaCl, 30 mM imidazole pH 8.0, 10% (vol/vol) glycerol], except for cultures expressing GyrA, which were resuspended in A800 [20 mM Tris base pH 8.0, 800 mM NaCl, 30 mM imidazole pH 8.0, 10% (vol/vol) glycerol], and sonicated using a Vibracell™ VCX500 ultrasonicator with medium tip (Sonics) for a total of 2 min (10 s on/10 s off). The sonicated sample was centrifuged at 20 000 × g for 1 h at 4°C to isolate the soluble fraction from cell debris. The protein rich isolated soluble fractions were passed through Ni-NTA His-Trap™ HP 5 mL columns (Cytiva) at 2 ml/min to maximise recombinant protein binding via N-terminal hexahistidine (6His) tags. A 10-column volume (cv) wash step was performed using lysis buffer. From this stage onward, purifications were optimised for each protein, detailed below. Fast protein liquid chromatography (FPLC) steps were carried out using an Åkta™ Pure protein chromatography system (Cytiva) at 4°C.

### Anion exchange chromatography

Protein samples were loaded on to a pre-equilibrated HiTrap Q HP anion exchange 5 ml column (Cytiva) in low salt buffer A100 [20 mM Tris base pH 8.0, 100 mM NaCl, 10% (vol/vol) glycerol]. This column was then subjected to an increasing salt gradient using the Åkta™ system, titrating in high salt buffer C1000 [20 mM Tris base pH 8.0, 1000 mM NaCl, 10% (vol/vol) glycerol] until a final salt concentration of 600 mM NaCl was achieved. 2 ml fractions were collected and analysed by SDS-PAGE. Fractions containing the protein of interest were carried forward for further purification or dialysed into an appropriate buffer for storage.

### Size-exclusion chromatography (SEC)

HiPrep 16/60 Sephacryl S-200 and S-300 HR SEC columns (Cytiva) were selected dependent on the column fractionation range and size of the target protein. The column was pre-equilibrated in sizing column buffer, S500 [50 mM Tris base pH 8.0, 500 mM KCl, 10% (vol/vol) glycerol], prior to a concentrated protein sample being applied via capillary loops at a rate of 0.5 ml/min. Fractionation occurred at 0.5 ml/min and the resulting chromatographic peaks were sampled and analysed by SDS-PAGE. Fractions containing the protein of interest were carried forward for further purification if needed, dialysed into an appropriate buffer, or stored.

### Purification and storage of *M. tuberculosis* GyrA

Once bound to the initial Ni-NTA and washed with 10 cv A800, the column was washed with a further 5 cv A100. The sample was eluted directly on to a pre-equilibrated anion exchange column with 10 cv B100 [20 mM Tris base pH 8.0, 100 mM NaCl, 250 mM imidazole pH 8.0, 10% (vol/vol) glycerol] before washing again in A100 to remove the high imidazole. The anion exchange column was run as above. Fractions were analysed for protein purity by SDS-PAGE, and appropriate fractions were pooled before the addition of 0.4 mg 6His-TEV protease to cleave the 6His-TEV site tag. The sample was rolled at 30 rpm in 4°C overnight then passed down a second Ni-NTA column (ortho Ni-NTA) to remove the 6His-TEV protease and 6His-TEV site tag. The flowthrough was collected and concentrated in a 10 kDa cut-off centrifugal concentrator (Sartorius) to 2 ml. The 2 ml sample was injected into a 2 ml capillary loop on the Åkta™ Pure system before fractionation by SEC using the Sephacryl S-300 column, as per above. Fractions were analysed for purity by SDS-PAGE, appropriate fractions were pooled and concentrated to >300 μM before diluting by one third volume with storage buffer [50 mM Tris base pH 8.0, 500 mM KCl, 70% (vol/vol) glycerol] for a final glycerol (cryoprotectant) concentration of 30%, and final protein concentration of >200 μM. Appropriate volume aliquots were made and flash cooled in liquid nitrogen before storage at −80°C.

### Purification and storage of *M. tuberculosis* GyrB

Once bound to the initial Ni-NTA and washed with 10 cv A500, the column was washed with a further 5 cv A100. The sample was eluted directly on to a pre-equilibrated anion exchange column with 10 cv B100 before washing again in A100 to remove the high imidazole. The anion exchange column was run as above. Fractions were analysed for protein purity by SDS-PAGE, and appropriate fractions were pooled before the addition of 0.4 mg 6His-TEV protease to cleave the 6His-TEV site tag. The sample was rolled overnight at 4°C then passed down a second Ni-NTA column (ortho Ni-NTA) to remove the 6His-TEV protease and 6His-TEV site tag. The flow through was collected and concentrated in a 10 kDa cut-off centrifugal concentrator (Sartorius) to 2 ml. The 2 ml sample was injected into a 2 ml capillary loop on the Åkta™ Pure system before fractionation by SEC using the S-300 column, as above. Fractions were analysed for purity by SDS-PAGE, appropriate fractions were pooled and concentrated to >300 μM before diluting by one third volume with storage buffer for a final glycerol (cryoprotectant) concentration of 30%, and final protein concentration of >200 μM. Appropriate volume aliquots were made, and flash cooled in liquid nitrogen before storage at −80°C.

### Purification and storage of *M. tuberculosis* ParDE1

This process was identical to production of GyrB with the following exceptions: tag cleavage occurred using the hSENP-2 enzyme (35) to remove the 6His-SUMO tag and SEC was performed using the Sephacryl S-200 column, as per above. Protein was stored in SEC buffer only (10% glycerol) at a concentration of >100 μM.

### Purification and storage of *M. tuberculosis* ParDE1 as a heterotetramer

This process was identical to production of GyrB with the following exceptions: tag cleavage occurred using the hSENP-2 enzyme to remove the 6His-SUMO tag concentration and SEC via the HiPrep 16/60 Sephacryl columns was not performed. Protein was of sufficient purity and concentration after anion exchange for biophysical studies.

### Purification and storage of *M. tuberculosis* ParDE2

Once bound to the initial Ni-NTA and washed with 10 cv A500, the sample was eluted in 5 cv B500 [20 mM Tris base pH 8.0, 500 mM NaCl, 250 mM imidazole pH8.0, 10% (vol/vol) glycerol] and 0.4 mg 6His-hSENP-2 was added to cleave the 6His-SUMO tag. The sample was dialysed into A100 overnight at 4°C before being passed down a second Ni-NTA column (ortho Ni-NTA) to remove the 6His-hSENP-2 and 6His-SUMO. The flow through was passed directly on to an anion exchange column for fractionation as above. Fractions were analysed for protein purity by SDS-PAGE; routinely ParE2 eluted in an early ‘shoulder’ peak before the full ParDE2 complex. Appropriately pure ParE2 fractions were not subjected to SEC due to low yields, rather, the sample was pooled and concentrated in a 10 kDa cut-off centrifugal concentrator (Sartorius) to >100 μM before flash cooling in aliquots for storage at −80°C.

### Gyrase assays

Gyrase assays were performed using published protocols (2,3), adapted where appropriate. The DNA gyrase holoenzyme was reconstituted by incubating equimolar amounts of GyrB and GyrA to a final heterotetramer (GyrB_2_A_2_) concentration of 10 μM on ice for 5 min. Gyrase fusion proteins were incubated at a final dimer concentration of 10 μM on ice for 5 min. Gyrase enzymes were then serially diluted in twofold steps using gyrase dilution buffer [50 mM Tris base pH 8.0, 2 mM MgOAc, 1 mM DTT, 500 mM KOAc, 50 μg/ml BSA, 10% (vol/vol) glycerol], down to the appropriate concentration for assays.

Each DNA relaxation reaction contained 5 μl of 4× gyrase reaction buffer [40 mM Tris base pH 8.0, 38.4 mM MgOAc, 4 mM DTT, 100 μg/ml BSA, 32% (vol/vol) glycerol)] and 1 μl of a 250 ng/μl solution of negatively supercoiled pSG483. 4 μl of the appropriate gyrase enzyme dilution was added before incubation on ice for 5 min. Reactions were then diluted to 20 μl with dH_2_O and incubated at 37°C for 30 min.

Each cleavage assay using TA components to interrupt gyrase DNA relaxation contained 5 μl of 4× gyrase reaction buffer and 1 μl of a 250 ng/μl solution of negatively supercoiled pSG483. 4 μl of 0.15625 μM gyrase enzyme (obtained by sequential two-fold dilutions of 10 μM stock) was added before incubation on ice for 5 min. 2 μl of protein dilution was added, or solvent/buffer where appropriate, before incubation on ice for a further 5 min. Reactions were diluted to 20 μl with 8 μl dH_2_O and incubated at 37°C for 30 min. Protein additive (TA system components and complexes) dilutions were prepared by two-fold dilution in respective storage buffers to appropriate assay concentrations.

Following incubation, reactions were first quenched with 2 μl of stopping buffer [5% (wt/vol) SDS, 125 mM EDTA], followed by adding 1 μl of 12 mg/ml proteinase K and further incubation at 37°C for 1 h. Reactions were stored at 4°C until immediately before gel loading, whereupon a 6× agarose gel loading dye was added to the samples and the samples were warmed to 37°C for 5 min. Samples were separated by electrophoresis in 1.4% (wt/vol) TAE agarose gels (containing 0.5 μg/ml EtBr as stated (when appropriate) for 16 h at 45 V. Agarose gels were post-stained in TAE containing 0.5 μg/ml EtBr (when appropriate) and visualised by UV illumination and were imaged on a BioRad ChemiDoc™ XRS + with ImageLab™ software on the EtBr setting (BioRad). Gel images were analysed using ImageJ2 (38) with background subtracted. For DNA relaxation assays, supercoiled band intensity was measured throughout the titration and converted to percentage of the ‘0′ gyrase lane supercoiled band. Cleavage assay measurements were taken from gels containing EtBr (when possible); supercoiled, linear, and nicked band intensities were calculated per lane. Linear band percentage was subsequently calculated per lane and normalised to the ‘0’ lane linear percentage, per assay. Measurements for the DNA damage induced by thermal remodelling of ParDE1 were performed on gels containing EtBr. Linear and nicked product estimates were calculated as per cleavage assays. To estimate the amount of DNA loss per lane the total band intensity of supercoiled + linear + nicked per lane was compared as a percentage to the band intensity of the control supercoiled (S) lane. The difference in percentage between the experimental lane and control lane is presented as DNA loss. Mean values and standard deviation were calculated from triplicate data (unless otherwise stated in figure legends) for the band of interest. Data were plotted in GraphPad Prism (Version 9.4.1) and presented with connecting line and error bars.

### Mass spectrometry

ES-TOF mass spectrometry of protein samples was kindly performed on the Xevo QtoF Premier mass spectrometer (Waters, UK) at our in-house Durham University Chemistry Department facility by Mr Peter Stokes. 100 μl protein samples were supplied at 1 mg/ml in 10 mM ammonium bicarbonate.

### Circular dichroism spectroscopy and thermal denaturation

Both circular dichroism (CD) and thermal denaturation were performed in-house on a J-1500 JASCO CD spectrometer. CD was performed at 20°C pre and post melting to analyse secondary structure of TA complexes. Thermal denaturation was performed between 20°C and 80°C with unfolding measured via the CD at 222 nm as a function of temperature. Proteins were analysed in A500 buffer. Spectra and melts were collected in a 1 mm pathlength cuvette with 1 nm data pitch on spectra and a thermal gradient of 1°C/min. The protein concentrations were 100 μM. Both CD and thermal denaturation curves are plotted in GraphPad Prism (Version 9.4.1) as an XY table, with X as ‘Numbers’ and Y as a ‘single Y value for each point’. Graphs are presented with the connecting line only. Melting temperatures were calculated using the JASCO thermal analysis software.

### Analytical SEC

The Superose 6 10/300 GL SEC column (Cytiva, discontinued) was selected for its broad fractionation range and short run time, allowing for analysis and purification on the Åkta™ pure system (Cytiva). Calibration curves were generated for the Superose 6 10/300 GL SEC column using appropriate combinations of commercially available low and high molecular weight kit proteins (Cytiva) for best resolution. The column was equilibrated in buffer S300-A [20 mM Tris base pH 8.0, 300 mM NaCl). For analysis, protein samples were manually loaded into a 100 μl capillary loop in their respective storage buffers at appropriate concentrations to generate a clear UV signal, generally 1 mg/ml was sufficient. Samples were injected onto the column using S300-A buffer at a flowrate of 0.5 ml/min for fractionation across 1.2 cv. Column volume, aka *V*_c_ in the equation below, was 24 ml. Where appropriate, samples were collected for further analysis in 250 μl fractions. Elution volumes (*V*_e_) were calculated using the Peaks function in Unicorn™ 7 (Cytiva). Elution volumes (*V*_e_) were converted into the partitioning coefficient (*K*_av_) for each sample using the following equation:


\begin{equation*}{K}_{av} = \ \frac{{{V}_e - {V}_o}}{{{V}_c - {V}_o}}\end{equation*}

The molecular weight calibration curve is subsequently plotted as *K*_av_ versus log_10_(*M*_r_, kDa). The Stokes radius (*R*_st_) calibration curve is subsequently plotted as log_10_(*R*_st_, Å) versus *K*_av_.

### Molecular weight (*M*_r_) and Stokes radius (*R*_st_) estimation

For estimates of *M*_r_ and *R*_st_, linear regression was performed on the respective plots. The resulting line equations (*y* = *mx* + *c*) were used to calculate the observed *M*_r_ and *R*_st_ through the following rearrangements:


\begin{equation*}{M}_{r} = 10\wedge \left( {\frac{{{K}_{av} - c}}{m}} \right)\end{equation*}

\begin{equation*}{R}_{st} = 10\wedge \left( {(m\left( {{K}_{av}} \right) + C} \right)\end{equation*}

Observed values were then compared to calculated values of *M*_r_ and *R*_st_ and presented as a ratio of calculated:observed. *M*_r_ values were calculated using the online ProtParam tool (Expasy) (39). *R*_st_ values were calculated using crystal structures and/or AlphaFold generated models using the HullRad calculator (Fluidic Analytics) (40).

### ParDE1 complex remodelling

ParDE1 expressed and purified as above provides the starting material (theoretical heterotetramer) for remodelling experiments. Once samples were ready for analysis they were subjected to analytical SEC as described above. For initial incubation and buffer alteration experiments, ParDE1 concentration remained at 2.5 mg/ml (∼62.5 μM). Incubation at 4°C was performed in the fridge, while 37°C and 45°C incubation was performed in a thermocycler. For concentration dependent studies, ParDE1 was concentrated in a 5 kDa cut-off centrifugal concentrator column (Sartorius) from 2.5 mg/ml and 100 μl sampled at the appropriate concentrations. For the 37°C time-course, ParDE1 concentration begun at 4 mg/ml (100 μM) to allow for coupling with cleavage assays. 100 μl was sampled at each time-point and subjected to analytical SEC. Incubation was controlled in a thermocycler.

### Mass photometry

ParDE1 expressed and purified as above provided the starting material (theoretical heterotetramer) for mass photometry experiments. A 5 ml ParDE1 sample at 5.2 mg/ml was incubated at 37°C with shaking at 180 rpm. At each time point, a 200 μl sample was snap frozen in liquid N_2_. Solution-phase mass determination of the ParDE1 species present in each sample was then performed using the TwoMP (Refeyn) mass photometer. Samples were diluted 1000-fold in A500, and experimental data were obtained in the form of mass photometry videos recorded for one minute using the AcquireMP v2.5 software (Refeyn) on precleaned, high sensitivity microscope slides. A mass calibration was done using bovine serum albumin, IgG, and thyroglobulin. The experimental data were then fit to this calibration, and graphs were generated using the DiscoverMP v2.5 software (Refeyn).

### Protein crystallisation

Samples for crystallography were dialysed into buffer X [20 mM Tris base pH 8.0, 150 mM NaCl, 2.5 mM DTT] and concentrated to 12 mg/ml (ParDE1) or 16 mg/ml (ParDE2) for initial trials. Sitting drop crystallisation trials were set-up using an SPT Labtech Mosquito® robot and commercial screens (Molecular Dimensions). Crystal screens were left at 18°C. ParDE1 required no optimisation, datasets of sufficient quality were collected from needle shaped crystals grown in 0.1 M Bis Tris Propane pH 7.5, 20% PEG 3350, 0.2 M NaNO_3_ and harvested directly from the crystal screen. Following an increase of starting concentration to 20 mg/ml, the best ParDE2 crystals (large hexagonal planar) grew after 3 months in 0.1 M MES pH 6.2, 15% wt/vol PEG 3350. For harvesting, mother liquor from the condition and 100% glycerol were mixed in a ratio of 1:1 and an equal volume of this mixture was added to the sitting drop, prior to looping and flash cooling in liquid nitrogen followed by storage in a puck for transport.

### X-ray data collection and processing

Data collection was performed at Diamond Light Source, Oxford, UK, via remote access on i04. Initial data processing was automated by Diamond Light Source iSpyB using the X-ray image integration programs Xia2 and Xia2-DIALS (41). Image integration and space group selection were carried out manually using the same programs as well as Mosflm (42).

For ParDE2, six, 360°, datasets were collected from two native ParDE2 crystals and merged using iSpyB (Diamond Light Source). For ParDE1, single datasets were collected from three native crystals and merged. Diffraction data were processed with XDS (43,44), and then AIMLESS from CCP4 (45) was used to corroborate the spacegroups. The crystal structure of ParDE2 was solved by molecular replacement using PHASER (46) and the *M. tuberculosis* H37Rv ParE2 AlphaFold structure prediction (47,48) as the search model. The crystal structure of ParDE1 was solved by molecular replacement using the starting model 3KXE (27) split into individual protomers ParD1 and ParE1 and input as individual assemblies. The solved starting models were built in REFMAC (49) and BUCCANEER (50). Initially, ParD2 could not be placed by PHASER. The ParE2 AlphaFold search model was edited to remove the C-terminal 12 amino acids to allow for subsequent manual building of the ParD2 chain in Coot (51). The models were then iteratively refined and built using PHENIX (52) and Coot, respectively. The quality of the final models was assessed using Coot and the wwPDB validation server (53). PyMol (Schrödinger) was used to perform sequence (‘align’ command) and structure-based (‘super’ command) alignments, and generate figures.

### Generation of AlphaFold multimer models

Protein structure predictions for the monomers of the TA system proteins are readily available in the published AlphaFold database, accessible online (48). For multimer models, the Google Colaboratory (ColabFold) (54) was used. This allowed for the input of multiple protein sequences and the subsequent automated generation of multimer models. Protein sequences for *M. tuberculosis* TA system components were sourced from Mycobrowser (55). The highest scoring models from these structure predictions are presented in this study.

## Results

### Differential toxicity and autoregulation of ParDE systems from *Mycobacterium tuberculosis*

**Figure 1.**
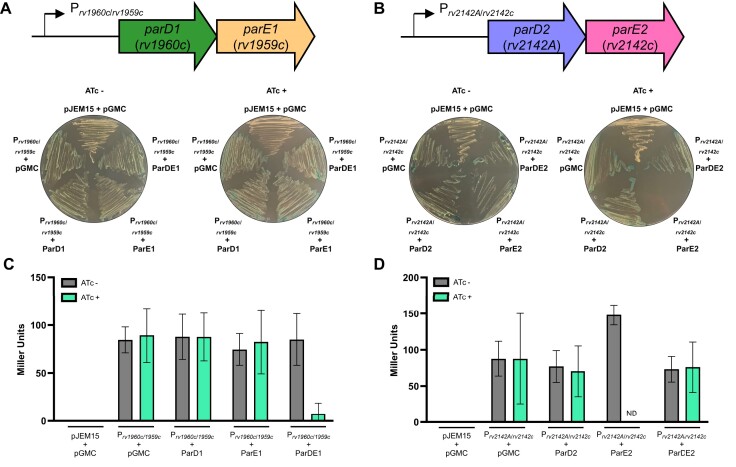
Toxicity and transcriptional autoregulation of *M. tuberculosis* ParDE systems. (**A**) and (**B**) Schematics of the *M. tuberculosis parDE* systems and co-transformants of *M. smegmatis* mc^2^155 containing the promoterless-*lacZ* pJEM15 vector-only and *parDE* promoter plasmids, together with the inducible pGMC vector-only or *parDE* expression plasmids, plated on LB plates containing X-gal (40 μg/ml) in the absence and presence of the pGMC inducer anhydrotetracycline (ATc, 100 ng/ml). ParE2 was toxic under these conditions. (**C**) and (**D**) β-galactosidase activity as determined from liquid culture of the above co-transformed strains. ND indicates no data obtained for the induced ParE2 condition, due to toxicity. Graphs show mean values, and error bars represent the SD of triplicate data. ParDE1 negatively autoregulated transcriptional activity.

*Mycobacterium tuberculosis* H37Rv encodes two *parDE* loci (Figures 1A and B). We began the study by examining both the toxicity of the ParDE systems and their capacity for transcriptional autoregulation, using *M. smegmatis* mc^2^155 (Figure 1). When expressing ParE1 or ParE2 toxins, only ParE2 was toxic in *M. smegmatis* (Figures 1A and B). Co-expression with antitoxin ParD2 restored growth (Figure 1B). Having cloned 1000 bp of upstream sequence from each *parDE* locus into the promoterless *lacZ* reporter plasmid pJEM15 (36), we observed that both promoters were active in *M. smegmatis* (Figures 1C and D). We then found that co-expression of both ParD1 and ParE1 caused negative autoregulation of transcription, but not with either component alone (Figure 1C). No autoregulation was demonstrated under these conditions for ParDE2 (Figure 1D).

### ParE toxins from *Mycobacterium tuberculosis* enhance gyrase-mediated DNA linearisation

As ParE toxins target DNA gyrase, we aimed to verify the activity of the *M. tuberculosis* toxins in gyrase assays. Having first purified independent GyrB and GyrA subunits of *M. tuberculosis* DNA gyrase, gyrase activity was confirmed by reconstituting the GyrB_2_A_2_ holoenzyme *in vitro* and testing for supercoil relaxation (Supplementary Figures S1A and B). *M. tuberculosis* gyrase successfully converted > 90% of the supercoiled pSG483 substrate into multiple topoisomers at a concentration of 31.25 nM, followed by decreased activity at saturating gyrase concentrations (Supplementary Figures S1A and B). The observed activity was comparable to previously demonstrated activity for *M. tuberculosis* gyrase (56).

Due to the toxicity of ParE1 in *E. coli*, genes *parE1* (*rv1959c*) and *parD1* (*rv1960c*) were cloned into a pET-Duet vector (57) for expression and purification of the ParDE1 protein complex (Supplementary Figure S2). Though we could not separate ParE1 and ParD1 during purification, the final ParDE1 complex did, however, form two peaks during size exclusion chromatography (SEC) (Supplementary Figure S2B). The final ParDE1 sample had high purity, as shown by SDS-PAGE and ES^+^-TOF mass spectrometry (Supplementary Figures S2C and D).

**Figure 2.**
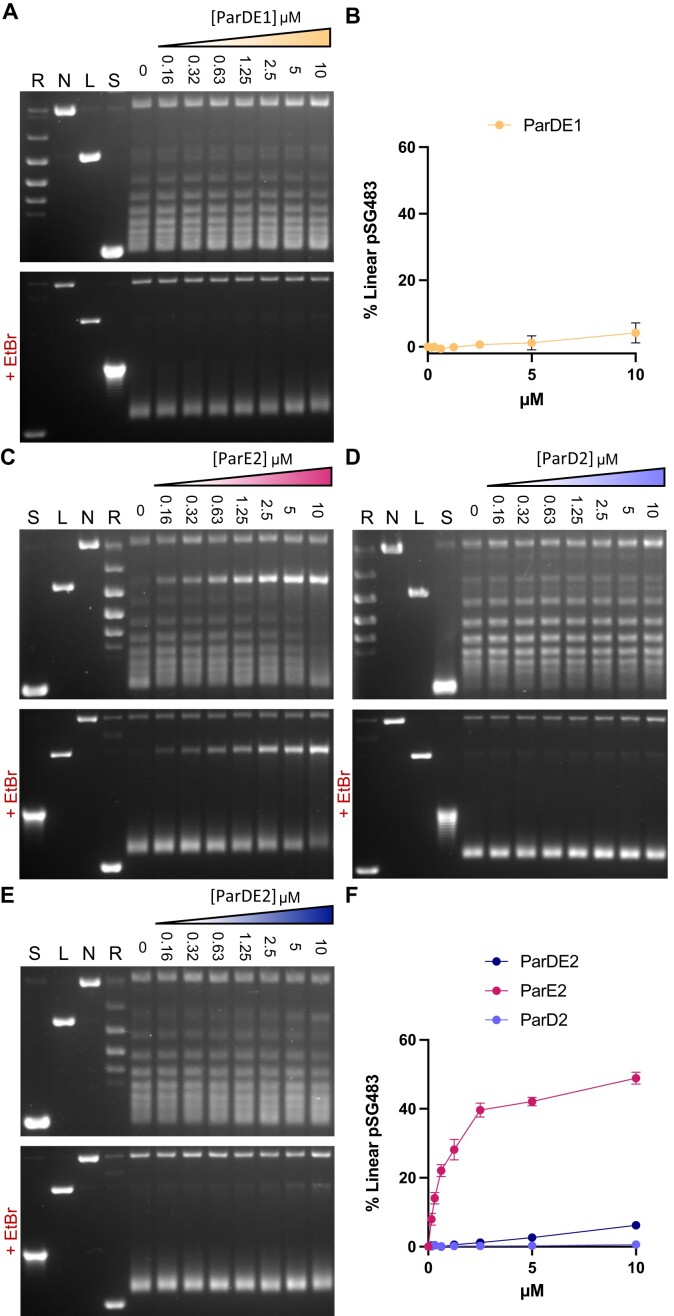
ParE toxins induce gyrase-mediated DNA linearisation. (**A**) ParDE1 induced DNA cleavage assay. (**B**) Linearisation of pSG483 as shown in (**A**). (**C**) ParE2 induced cleavage assay. (**D**) ParD2 induced cleavage assay. (**E**) ParDE2 induced cleavage assay. (**F**) Linearisation of pSG483 as shown in (**C**-**E**). ParDE system components were titrated against constant GyrB_2_A_2_ (31.25 nM) and Supercoiled (S) plasmid DNA (12.5 nM). ParDE protein concentration per lane is presented below the agarose gels. Control lanes represent Supercoiled (S), Linear (L**)**, Nicked (S) and Relaxed (multiple topoisomers) (R) plasmid DNA. Assays are presented on 1.4% agarose 1× TAE gels (run with ethidium bromide (+EtBr) as stated, or post-stained). Assays shown are representative of triplicate experiments, and data points and error bars represent the mean and SD of triplicate data.

In the absence of free ParE1 toxin for assays, the ParDE1 complex was nevertheless tested in a gyrase DNA relaxation assay (Figures 2A and B). At the highest concentration tested (10 μM), ParDE1 caused a small amount (∼4%) of linearisation (Figures 2A and B). This suggested that over the course of the assay a small amount of the ParE1 toxin had potentially been released, and could trap gyrase complexes.

Genes *parD2* (*rv2142A*) and *parE2* (*rv2142c*) were then sequentially cloned into a pET-Duet vector (57) allowing for co-expression and purification of the ParDE2 protein complex, or ParD2 alone (Supplementary Figure S3A). During purification of ParDE2 it was found to also be possible to separate and purify some ParE2 toxin away from the complex (Supplementary Figure S3B). During anion exchange of the cleaved ParDE2 complex, ParE2 was isolated in a distinct chromatographic peak. SDS-PAGE analysis showed the purified 12.18 kDa ParE2 toxin alongside the ParDE2 complex (Supplementary Figure S3C). ES^+^-TOF mass spectrometry of the purified ParE2 sample showed there was no detectable ParD2 in the sample (Supplementary Figure S3D).

The purified ParDE2 samples were then tested against DNA gyrase (Figures 2C–F). ParE2 was tested first, using an *M. tuberculosis* gyrase DNA relaxation reaction (Figure 2C). At the highest concentration tested, ParE2 caused linearisation of ∼50% of substrate pSG483 (Figures 2C and F), as normalised against a toxin-only control (Supplementary Figure S1C). In contrast, purified ParD2 generated no significant increase in linear pSG483 in the gyrase DNA relaxation reaction (Figures 2D and F). The purified ParDE2 complex was also tested in the gyrase DNA relaxation reaction (Figures 2E and F). The ParDE2 complex, like ParDE1, caused ∼5–7% linearisation at the highest concentrations of 5 and 10 μM ParDE2, respectively (Figures 2E and F). Collectively, these results demonstrate the ability of the ParE2 toxin to inhibit DNA gyrase by trapping the cleavage complex, causing the persistence of linearised DNA.

### ParD2 displaces the ParE2 C-terminal helix to occupy a conserved hydrophobic surface patch

Our purification and *in vitro* data suggested that *M. tuberculosis* ParE toxins can be released from ParDE complexes. Next, we performed structural studies to understand the interactions within the ParDE1 and ParDE2 complexes.

**Figure 3.**
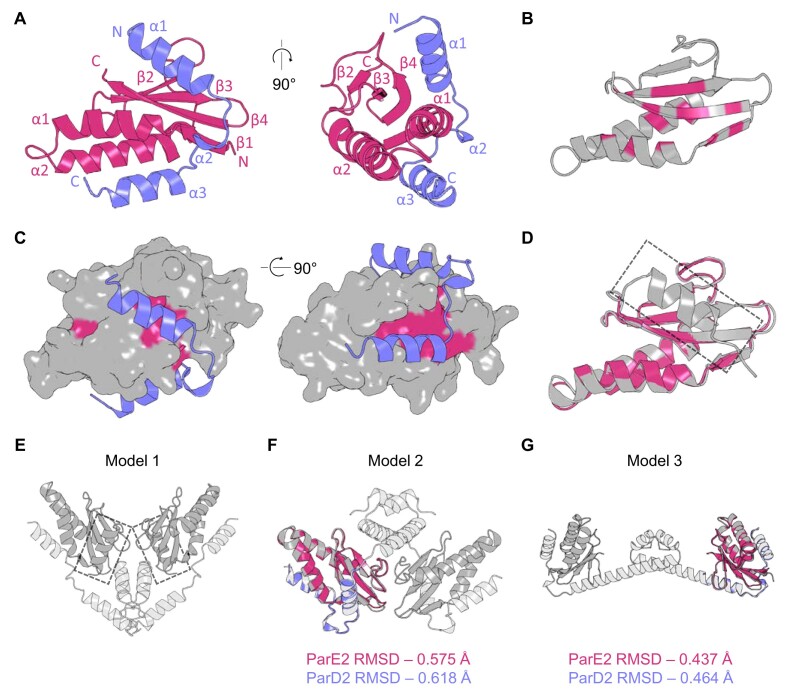
ParD2 displaces the C-terminal helix of ParE2. (**A**) Cartoon representation of the dimeric ParDE2 complex crystal structure with 90° rotation. ParE2 is coloured magenta, ParD2 is coloured slate blue. (**B**) Cartoon representation of the ParE2 toxin, coloured grey, with conserved residues from the RelE/ParE superfamily highlighted in magenta. (**C**) Surface rendered ParE2 with conserved residues in magenta complexed with ParD2 (coloured slate blue). (**D**) Sequence-based alignment of AlphaFold ParE2 (now grey) to the crystal structure of ParE2 (magenta) returned an RMSD of 0.594 Å. Additional C-terminal helix of the AlphaFold model is indicated by the dashed box. (**E**) AlphaFold multimer ‘Model 1′ of ParD2_2_ParE2_2_, with ParE2 protomers in dark grey and ParD2 protomers in light grey. Additional C-terminal helices of ParE2 are indicated by the grey boxes either in front (left) or behind (right) the complex. (**F**) AlphaFold multimer ‘Model 2′ of ParD2_2_ParE2^Δ88–105^_2_, with ParE2 protomers in dark grey and ParD2 protomers in light grey. The ParDE2 crystal structure is aligned to the AlphaFold model. RMSD values for the individual ParE2/ParD2 alignments are presented below. (**G**) AlphaFold multimer ‘Model 3′ of ParD2_2_ParE2^Δ88–105^_2_, with ParE2 protomers in dark grey and ParD2 protomers in light grey. The ParDE2 crystal structure is aligned to the AlphaFold model. RMSD values for the individual ParE2/ParD2 alignments are presented below.

ParDE2 was our first structural target. Antitoxin ParD2 and toxin ParE2 were co-expressed and purified as described earlier and put into crystallisation trials. After three months of growth, the resulting crystals were used to collect X-ray diffraction data. The phase problem was solved by molecular replacement using a truncated ParE2 (amino acids T2-G86) AlphaFold model (47) as a search model, and ParD2 (amino acids I36-G71) was then built into the remaining electron density. The final model was refined to 2.35 Å (Figure 3A, Table 1).

**Table 1.** X-ray data collection and refinement statistics

	ParDE2	ParDE1
PDB ID code	8C26	8C24
Number of crystals	1	3
Beamline	Diamond I04	Diamond I04
Wavelength, Å	0.9793	0.9795
Resolution range, Å	50.59–2.35 (2.429–2.35)	45.92–2.10 (2.175–2.10)
Space group	*R* 3 2 : *H*	*P* 1 21 1
Unit cell, *a b c* (Å), α β γ (°)	68.074 68.074 197.049, 90 90 120	44.56 125.45 52.26, 90 90 90
Total reflections	160 967	207 238
Unique reflections	7244 (349)	31 537 (3147)
Multiplicity	19.8	1.9
Completeness (%)	100 (100)	99.58 (99.05)
Mean I/sigma(I)	7.5 (0.81)	9.13
*R*_merge_	NA	0.036 (0.478)
*R*_meas_	NA	0.050 (0.676)
CC_1/2_	1.0	0.999 (0.669)
*R*_work_	0.2539 (0.5053)	0.198 (0.297)
*R*_free_	0.2883 (0.5020)	0.234 (0.333)
No. of non-hydrogen atoms	1023	3634
Macromolecules	1021	3519
Ligands	2	18
Solvent	0	97
Protein residues	123	440
RMSD (bonds, Å)	0.008	0.007
RMSD (angles, °)	0.99	0.78
Ramachandran favoured (%)	85.71	98.36
Ramachandran allowed (%)	14.29	1.64
Ramachandran outliers (%)	0.00	0.00
Average *B*-factor	102.12	47.62
Macromolecules	102.09	47.48
Ligands	115.84	58.82

Values in parenthesis are for the highest resolution shell.

The ParDE2 crystal structure exists as a heterodimer containing a half-unresolved ParD2 antitoxin (Figure 3A, Supplementary Figure S4A). The ParE2 toxin is comprised of β1 (R4–H8), α1 (N9–Y22), α2 (P27–Q47), β2 (R60–Y63), β3 (Y69–T75), β4 (A79–H87), with the C-terminal M88–E105 being unresolved (Figure 3A, Supplementary Figure S4A). β1/α1 and α2 form a hairpin structure which links to the anti-parallel β sheet core of β2–β4. The β sheet core sits on top of the hairpin with β4 and β1 interacting in parallel (Figure 3A, Supplementary Figure S4A). The ParD2 antitoxin is comprised of α1 (E38–N49), α2 (D53–H55), α3 (I59–R69), with the N-terminal V2–H35 being unresolved in the crystal structure (Figure 3A, Supplementary Figure S4A).

A RelE/ParE superfamily alignment (25) allowed us to plot and visualise the conserved hydrophobic residues within the ParE2 toxin structure, which are clearly concentrated on the internally facing residues of the α helical hairpin and throughout the β sheet core of the toxin (Figure 3B). This positioning creates two major hydrophobic grooves on the surface of the ParE2 toxin which run along the β sheet core and through to the underside of the toxin between the hairpin helices (Figures 3B and C). Representing the ParD2 antitoxin alongside a surface rendered ParE2 toxin clearly demonstrates the specific interaction at the conserved hydrophobic patches, as they align closely to the truncated region of ParD2 (Figure 3C). This mimics the conserved mechanism of protein recognition identified in the *C. crescentus* ParDE structure (27). PISA analysis (58) of the ParDE2 complex highlights several polar and ionic interactions that stabilise the largely hydrophobic interfacing. Notable contacts are the ionic bonds formed between ParD2 E45 (on α1) and ParE2 K57 (found on the loop region between α2 and β2) (Supplementary Figure S4B) and between ParD2 R47 (on α1) and ParE2 (D14) (on α1, also) (Supplementary Figures S4C and D). These are highly specific side chain interactions that demonstrate the mechanism of ParDE2 interaction extends beyond a conserved hydrophobic groove.

A truncated AlphaFold ParE2 model was used for molecular replacement. In a full-length AlphaFold model of ParE2 the C-terminal P92 – G101 region forms additional helix α3, which occupies the interface for ParD2 binding that is bound by ParD2 α1 in the crystal structure (Figure 3D). Phasing the ParDE2 dataset is successful when the ParE2 C-terminal helix is included in the search model, but the linker region between β4 and the additional α3 is not resolved. In contrast, when the C-terminal helix of ParE2 is removed from the search model, unmodelled electron density is present in its place, and can be more successfully built as residues I36–N49 of ParD2. This demonstrated that ParD2 α1 displaced the predicted ParE2 α4, which likely became disordered as is seen for other ParDE system complexes.

AlphaFold multimer (59) was then used to generate a series of models of ParDE2 complexes. It was expected that, given the crystal structure data indicating the presence of the ParD2 α1 helix across the β sheet hydrophobic region, AlphaFold would generate a multimer model demonstrating preference for this interaction over the ParE2 α4 helix. This was not the case when the full ParE2 sequence was entered alongside the full ParD2 sequence; the full ParE2 model was generated with the additional C-terminal helix and the corresponding ParD2 helix displaced (Figure 3E). In this predicted model, the ParD2-ParE2 heterodimers are seen interacting through the bundled helices of ParD2 at loop regions. Based on our structure this model does not appear biologically relevant. We therefore investigated whether truncating the ParE2 sequence as input for AlphaFold multimer would present alternative solutions. Using the sequence for ParE2^Δ88–105^, with the unresolved C-terminal residues M88 – E105 deleted, AlphaFold generated two alternative complex models with a more biological quaternary structure (Figures 3F and G). Both these models have the ParD2 antitoxin occupying both hydrophobic patches across ParE2 and provide good structural alignments with the solved ParDE2 complex (Figures 3F and G, Supplementary Figures S4E and F). Additionally, in both models the ParD2 protomers now interact in the same way, via their N-terminal helices (Figures 3F and G), which is typically seen for members of the RelE/ParE TA system family (27,60–63).

We used analytical SEC data and Stokes radius (*R*_st_) calculations (40) (Supplementary Figures S3A and B, Supplementary Figure S4G) to determine the most likely model for ParDE2 between Models 2 and 3 (Figures 3F and G). The *R*_st_ for Model 2 (Figure 3F) was calculated to be 26.10 Å, and the *R*_st_ for Model 3 (Figures 3G) was calculated to be 30.50 Å (Supplementary Figure S4G). Model 3 provided a closer value to the observed *R*_st_ calculated from analytical SEC data (30.27 Å), indicating that this is our best model for the ParDE2 heterotetrameric complex.

Finally, given the time taken to crystallise, and that there is no remaining space in the crystal due to packing, it was considered likely that ParDE2 underwent limited proteolysis during crystallisation. This was confirmed by dissolving ParDE2 crystals and analysing by SDS-PAGE (Supplementary Figure S4H).

### ParDE1 forms a heterohexameric complex

**Figure 4.**
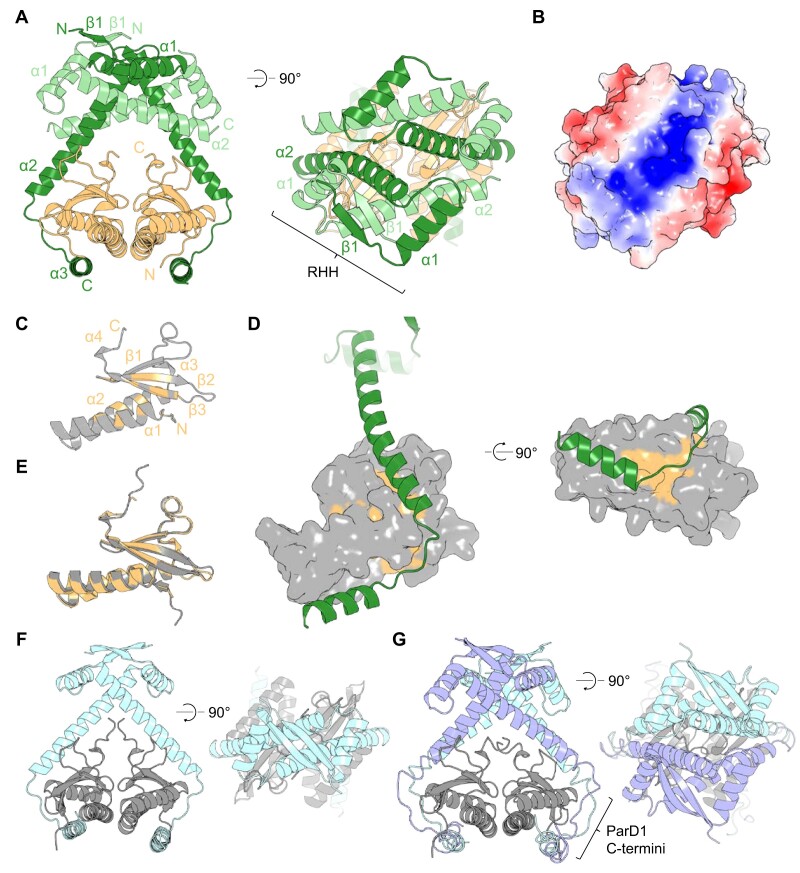
ParDE1 forms a heterohexameric complex. (**A**) Cartoon representation of the heterohexameric ParD1_4_ParE1_2_ complex crystal structure. ParD1 antitoxins exist in two forms within the structure; two full-length (primary) chains are coloured dark green with N and C termini labelled, and two short-length (auxiliary), partially resolved chains are coloured pale green, with N and C labelled. The two ParE1 toxins are coloured light orange with N and C termini labelled. The ParD1 tetramer is formed through a beta sheet between the primary and auxiliary antitoxins and creates a cage-like structure around the toxins. Rotated view (right) shows the N-terminal dimerisation domain of the ParD1 antitoxins forming a ribbon-helix-helix structure (RHH). (**B**) Surface rendering of the ParDE1 complex crystal structure coloured by electrostatic potential using the APBS plugin (PyMol). (**C**) Cartoon representation of the ParE1 toxin, coloured grey, with conserved residues from the RelE/ParE superfamily highlighted in light orange. (**D**) Surface rendered ParE1 with conserved residues in light orange complexed with Primary ParD1 (coloured forest green). (**E**) Sequence-based structural alignment of the ParE1 crystal structure (light orange) and AlphaFold model (dark grey), which returned an RMSD of 0.594 Å. (F and G) AlphaFold models of ParD1_2_ParE1_2_ heterotetramer (**F**) and ParD1_4_ParE1_2_ heterohexamer (**G**). Rotated view shows the ParD1 complexing region. AlphaFold models shown with ParE1 in dark grey and ParD1 in light blue and lilac.

Having determined the structure of the ParDE2 complex, we moved on to ParDE1. Antitoxin ParD1 and toxin ParE1 were co-expressed and purified as described earlier (Supplementary Figure S2). Following trials and optimisation, ParDE1 crystals grew as needles. Single datasets were collected from 3 crystals and subsequently merged to a final resolution of 2.10 Å. The ParDE1 complex structure was determined by molecular replacement using the *C. crescentus* ParDE structure, PDB: 3KXE, (27) as a search model (Figure 4A, Table 1). Interestingly, whilst phasing was successful, on viewing the unrefined density it was clear that a significant portion of the structure remained unmodeled. As a result, an additional two ParD1 chains were built into the model.

The ParDE1 complex revealed in the crystal structure is of a ParD1_4_ParE1_2_ heterohexamer, with two full-length (‘primary’) ParD1 antitoxins resolved alongside two shorter, partially resolved (‘auxiliary’), ParD1 antitoxins (Figure 4A, Supplementary Figure S5A). The ParE1 toxin is comprised of α1 (P9–W26), α2 (V28–A47), α3 (P49–I51), β1 (R61–A67), β2 (H70–T77), β3 (G80–H89) and α4 (Q90–M92), with 3 C-terminal residues unresolved (Figure 4, Supplementary Figure S5A). α1 and α2 form a hairpin-like structure as a base. The three beta strands form an antiparallel sheet that sits above the hairpin, followed by a short helix (α4). The primary ParD1 protomers are comprised of β1 (T5–V8), α1 (E13–A23), α2 (A29–S60), α3 (F68–S80). The auxiliary ParD1 protomer secondary structures are the same as the primary ParD1 protomers from residues G2–E55, with the C-terminal A56–R83 unresolved (Supplementary Figure S5A). There is variation in the pairs of ParD1 protomers; β1 is not resolved in one of each of the primary and auxiliary chains (Figure 4A).

The heterohexameric structure somewhat resembles the search model, with the ParD1 antitoxins interacting via an anti-parallel N-terminal β sheet (though noting only one pair of the antiparallel β sheets was resolved), and a pair of ParE1 toxins positioned inside a cage-like structure (Figure 4A). In the *C. crescentus* ParDE structure the ParD antitoxins interact through an additional coiled-coil between corresponding ParD1 α2 helices (27). In contrast, a coiled-coil structural motif is not seen between the primary ParD1 chains in the *M. tuberculosis* ParDE1 structure, rather, the two primary chains appear to lean against one another. A coiled-coil interaction is present, however, between helix α2 of the primary ParD1 chains and helix α2 of the auxiliary ParD1 chains, forming alongside the anti-parallel β sheet that is itself part of a ribbon-helix-helix (RHH) DNA binding motif (Figure 4A). Dali searches (64) using the ParD1 tetramer indicated structural similarity to transcriptional regulator CopG, localised to the dimerised N-terminus and RHH region, and aligning with RMSD values of <2.0 Å (PDB: 6IYA (65); 1EA4 (66)). This indicates that ParD1 belongs to the CopG/Arc/MetJ family (67). Surface electrostatics also show a large electropositive patch created at the antitoxin complexing region (Figure 4B). Using structures of *S. agalactiae* CopG (66,67), we can model the ParD1 tetramer interaction with DNA whereby the antiparallel beta sheets insert into the major groove of bent DNA (Supplementary Figure S5B). This proposed model accounts for how ParDE1 can perform negative transcriptional autoregulation (Figure 1C).

PISA analysis (58) of the ParDE1 heterohexamer identified 4 key interfaces (Supplementary Figure S6). The essential interfaces are at the antitoxin antiparallel β sheet and the primary ParD1:ParE1 interface, suggesting these might form more readily before higher order assembly (Supplementary Figure S6). Again using the RelE/ParE superfamily multiple sequence alignment (25) we noted the positions of conserved residues on the ParE1 surface (Figure 4C). As for ParE2 (Figure 3B), conserved residues within ParE1 are concentrated on the internal facing portions of α1 and α2, and along β2 and β3 (Figure 4C). Mapping these to the surface of the ParE1 toxin shows two highly conserved hydrophobic patches used as binding sites for a single ParD1 primary chain; one site along the groove created by the antiparallel β strands (1–3), and the other site on the underside of the toxin structure between the hairpin of α helices 1 and 2 (Figure 4D), as also picked out by PISA (Supplementary Figure S6). ParD1 antitoxin binds to these patches similarly to how ParD2 was observed binding ParE2 (Figure 3C), though it is notable that ParE1 does not encode the proposed C-terminal helix in ParE2 that is displaced by ParD2. ConSurf analysis shows complementary localisation of highly conserved residues between ParD1 and ParE1 (Supplementary Figure S7).

The absence of a C-terminal helix in ParE1 is supported by the AlphaFold model, which aligned to the ParE1 crystal structure with an RMSD of 0.594 Å (Figure 4E). We used AlphaFold multimer (59) to assess how stoichiometry might influence the overall quaternary structure. Looking more closely at data collected during ParDE1 purification strongly indicated the presence of two complexes in solution, the determined 4:2 complex structure, and a slightly smaller complex (Supplementary Figure S2B). This smaller peak might correspond to a more canonical 2:2 ParDE complex. Interestingly, as per our prediction, a 2:2 heterotetrameric complex could be generated by AlphaFold, creating a quaternary structure as per the search model used in MR (PDB: 3KXE) (Figure 4F). AlphaFold also successfully generated a 4:2 complex as per the crystal structure (Figure 4G) with the complexed regions of the antitoxins displaced off-centre creating the tetrameric CopG RHH. Interestingly, the AlphaFold 4:2 complex model presents the C-terminal ParD1 E55 – R83 regions as loosely structured in all 4 ParD1 monomers, perhaps due to perceived direct competition between what we have identified as the ‘primary’ and ‘auxiliary’ for the ParE1 surfaces (Figure 4G). Each of the C-terminal regions track along the ParE1 interface and form a weak helical structure resembling α3, however due to this direct competition neither is presented as fully folded. This indicates error in this AlphaFold solution, as the crystal structure shows only the primary ParD1 monomers form the interface.

### ParDE1 exists in a dynamic equilibrium between complexes

**Figure 5.**
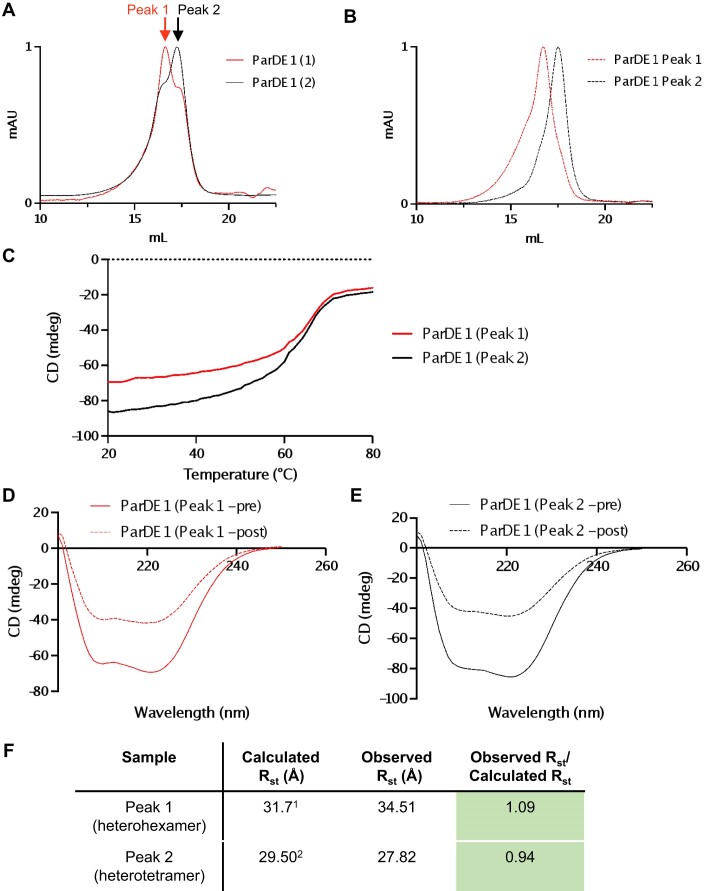
ParDE1 can form multiple complexes. (**A**) Analytical SEC traces of two independent purifications of the ParDE1 complex. (**B**) Analytical SEC traces of separated ParDE1 peaks from a mixed sample as presented in (A). (**C**) Protein thermal denaturation curves for the separated ParDE1 samples normalised to 222 nm. (**D**) Circular dichroism spectroscopy scans for ParDE1 peak 1, before (solid red) and after (dashed red) melting. (**E**) Circular dichroism spectroscopy scans for ParDE1 peak 2, before (solid black) and after (dashed black) melting; (**F**) Table of ParDE1 Stokes Radius (R_st_) calculations, observations, and comparisons. Comparison of observed/calculated is coloured green if within 10% of the predicted ratio, yellow if > 10 ≥ 25%, and red if > 25%. ^1^*R*_st_ for the crystal structure solution of ParD1_4_ParE1_2_; ^2^*R*_st_ for the AlphaFold solution of ParD1_2_ParE1_2_. *R*_st_ values were generated using HullRad (Fleming and Fleming, 2018). Chromatograms are representative of duplicate data and are normalised between 0 and 1 for presentation and comparison. Graphs are cropped to the appropriate scale (10–22.5 ml).

The obtained ParDE1 structure, purification data, and supporting AlphaFold results (Figure 4, Supplementary Figure S2B) suggested ParDE1 can form multiple quaternary complexes in solution. We noted that analytical SEC of two independent ParDE1 purifications produced distinct, but overlapping, UV traces containing two peaks (Figure 5A). Both peaks contained high purity protein of the appropriate sizes for ParD1 and ParE2. Conversion of the respective V_e_ to M_r_ indicated a complex of 84.22 kDa to be dominant in sample ParDE1 (1) (Figure 5A, red trace, ‘peak 1’) and a complex of 43.77 kDa to be dominant in sample ParDE1 (2) (Figure 5A, black trace, ‘peak 2″). Fractions corresponding to each of the noted peaks (Figure 5A) were then pooled, and each peak was analysed by SEC using a Superose 6 10/300 GL column (Figure 5B). Both peaks maintained different sizes and did not appear to re-distribute to multiple complexes under these conditions, and so represented purified ParDE1 complexes of distinct stoichiometries.

Separation of these two peaks allowed for thermal stability and circular dichroism analysis of the respective protein complexes (Figure 5C–E). Thermal denaturation analysis (melting) (68) showed that both samples had high thermal stability with melting temperatures of around 65°C (Figure 5C). The melting curves do not show complete unfolding as the CD signal ceases to change significantly above around 70°C. This is indicative of aggregation occurring as the samples unfold. The two samples show differences both in melting temperature, with the Tm estimated from the point of inflection in the melt curve being a few degrees lower for the peak 2 (black curve) than for the peak 1 (red curve), and with the loss of structure during aggregation being more significant for the peak 2 sample (Figure 5E) than for the peak 1 sample (Figure 5D). These differences further indicate that two different ParDE1 complex species are present, one from each peak.

To determine likely solution states for the complexes in each SEC peak, *V*_e_ values for both peaks in the same trace (Figure 5A) were used to estimate the *R*_st_ for the dominant ParDE1 species (Figure 5F). The observed *R*_st_ for peak 1 was 34.51 Å, and the observed *R*_st_ for peak 2 was 27.82 Å (Figure 5F). Using the earlier AlphaFold model (Figure 4F) supports peak 2 containing ParDE1 heterotetramer complexes, as the calculated *R*_st_ value of 29.50 Å agrees well with the observed R_st_ value of 27.82 Å (Figure 5F). Having identified peak 2, we proposed that peak 1 contained the heterohexameric complex we observed in our X-ray structure. Using our structure, the calculated *R*_st_ was 31.7 Å, which matched well with our observed *R*_st_ for peak 1 (Figure 5F). This supports peak 1 containing ParDE1 heterohexamer complexes. Taken together, these data strongly support a model wherein ParDE1 can exist in multiple stoichiometries in solution.

### Conditional remodelling of ParDE1 complexes

Interestingly, the independent purifications of ParDE1 show different relative intensities between the two complex species present (Figure 5A). This indicates a potential equilibrium between the two species and that one may become the other. We chose to explore the conditions that might impact this equilibrium.

**Figure 6.**
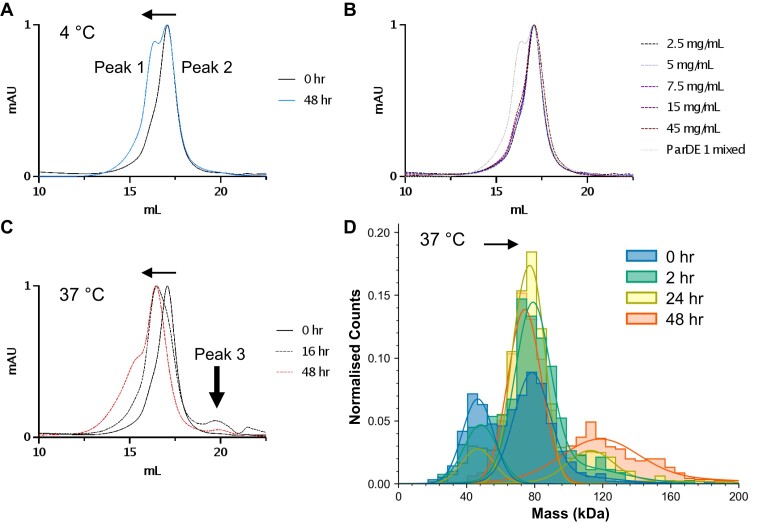
ParDE1 undergoes conditional complex remodelling. Analytical SEC traces for ParDE1 (**A**) Purified in the heterotetrameric fraction (0 hr, black). When incubated at 4°C this single peak shifts into the mixed peak (48 hr, blue). Positions of peak 1 and peak 2 are indicated. (**B**) Increasing complex concentration presented alongside the mixed sample from A (light grey). (**C**) 37°C incubation alongside the starting, 0 h trace (solid black). Vertical arrow indicates potential free ParE1 ‘peak 3″. Chromatograms are representative of single repeats and are normalised between 0 and 1 for presentation and comparison. Graphs are cropped to the appropriate scale (10–22.5 ml). (**D**) Mass photometry analysis demonstrates that, during incubation at 37°C, ParDE1 heterotetramer complexes (measured at mean 47–48 kDa) proportionally shift to heterohexameric complexes (measured at mean 74–81 kDa). At longer time points 24 and 48 h, larger species, potentially indicating higher order complexes, are observed (measured at mean 115–121 kDa). Graph shows normalised counts from approximately 1000–1500 molecules measured per data collection.

Having developed a method to isolate the ParDE1 heterotetramer (see Materials and Methods), the sample was subjected to a range of conditions (Figure 6, Supplementary Figure S8) to explore whether ParD1_2_ParE1_2_ can be remodelled into the predicted ParD1_4_ParE1_2_, as signified by the emergence of ‘peak 1′ from ‘peak 2′ during analytical SEC. Should this occur, we hypothesised that the ParE1 toxin might become free in solution. High-yield purification of ParDE1 (>50 ml at 2.5 mg/ml) in the heterotetramer stoichiometry (Figure 6A, black curve/0 h) allowed for the exploration of conditions that may influence protein complex states, notably ParDE1 concentration, temperature, pH, and salt concentration. Following incubation at 4°C for 48 h, the chromatographic trace does indeed change and the heterohexameric ParDE1 complex ‘peak 1′ grows from the original heterotetrameric ‘peak 2′ observed at 0 h (Figure 6A).

The starting material, at 2.5 mg/ml (or 0 h) was then concentrated and sequentially analysed via SEC (Figure 6B). The 48 h ‘mixed’ ParDE1 sample (Figure 5A) is presented alongside the increasing concentrations to help identify the respective ‘peaks’. It is clear to see that concentrating ParDE1, even as high as 45 mg/ml, does not have a large effect on the predicted stoichiometries; as, at each concentration, the dominant species remains aligned with the heterotetrameric (peak 2) starting material at 2.5 mg/ml. There is a minor shift at the higher concentrations with the appearance of a ‘shoulder’ that aligns with the heterohexameric ‘peak 1’. Considering the appearance of peak 2 over time (Figure 6A), it is possible that the shoulder is an artefact of the experiment as concentration of the sample and the sequential analytical SEC analysis took over 6 h.

As the heterohexamer appears to be around 5°C more thermostable than the heterotetramer via melt analysis (Figure 5C), it was hypothesised that complex remodelling may be thermodynamically driven. Incubating ParDE1 heterotetramer at a starting concentration of 2.5 mg/ml at 37°C results in a more rapid evolution of the heterohexameric peak 1 (Figure 6C, 16 h). Not only is the emergence of the peak apparently more rapid than in the 4°C incubation over 48-hours, it is also more dominant in terms of the respective intensities of the two peaks, with the heterohexamer being the dominant species in 37°C incubation. At the 48-hour time-point, not only has the entire sample shifted from the heterotetramer to the heterohexamer, but a shoulder appeared on the left of the heterohexamer, indicating that the entire fraction has been remodelled into at least heterohexamer and perhaps even higher order complexes (Figure 6C, 48 h). Crucially, an additional small peak was observed just after 20 ml elution, most evident on the 16-h curve, which could be formed by free ParE1 toxin (Figure 6C, Peak 3), as previously hypothesised. A higher temperature of 45°C was then selected as a further test condition, which sped up the complex remodelling futher (Supplementary Figure S8A). Again, a potential ParE1 peak was obtained (Supplementary Figure S8A, Peak 3). Next, we briefly explored the effects of reducing agent, acidic pH, and high salt on remodelling, as these conditions can all effect protein complex states and are environmental conditions to which TA systems may be responsive (17). None of these conditions caused any noticeable shift in the positioning of the starting peak (Supplementary Figure S8B).

Having established the positions of three chromatographic peaks of interest (peak 1 – heterohexamer; peak 2 – heterotetramer; peak 3 – ParE1), fractionation and SDS-PAGE analysis was used to examine potential purification of the respective species (Supplementary Figure S8C). Both proteins, ParE1 (11.17 kDa), and ParD1 (9.21 kDa), were present in each fraction (Supplementary Figure S8C). Peak 3 overlays with the tail of the heterotetramer peak, which accounts for the presence of ParD2 in this region of the chromatogram. Nevertheless, the AlphaFold-predicted ParE1 structure produced a calculated R_st_ of 16.40 Å. The calculated R_st_ of peak 3 was 15.82 Å, providing an observed/calculated ratio of 0.96 (Supplementary Figure S8D), indicating that the peak produced during the hypothesised ParDE1 remodelling process could theoretically be free monomeric ParE1. However, this did not appear to be a suitable method for purification of the desired ParE1 toxin due to the very small quantities of free ParE1 protein produced. We attempted to release and purify ParE1 on a larger scale via incubation at higher temperature. A ParDE1 sample was concentrated to 10 mg/ml and incubated at 37°C for 16 h, prior to SEC. Unfortunately, post-incubation a high amount of precipitate was present. Following centrifugation, the supernatant was analysed by SEC and the chromatographic trace was aligned to previous ParDE1 purification, indicating the sample had re-modelled to heterohexamer but no ParE1 peak was obtained (Supplementary Figure S8E). The major peak fraction was analysed alongside the precipitate fraction from overnight incubation (Supplementary Figure S8F). This demonstrated that SEC produced purified ParDE1 complex (Supplementary Figure S8F, ParDE1 lane), whereas the liberated ParE1 toxin after 37°C incubation appears to have precipitated (Supplementary Figure S8F, ParE1 lane). Though unfortunate, the appearance of ParE1 precipitate supports our hypothesis for ParE1 release as a result of complex remodelling.

Next, we performed mass photometry analysis of ParDE1 samples incubated at 37°C to provide an additional biophysical demonstration of complex remodelling (Figure 6D). Within two hours there was a substantial shift from heterotetramers to majority heterohexamers (Figure 6D). At later time points (24 and 48 h) we observed a population of larger molecules that may represent higher order ParDE1 complex structures (Figure 6D). These would correspond with the additional shoulder observed at 48 hr incubation by SEC (Figure 6C). These two pieces of data are thought unlikely to represent aggregates, as aggregates would appear in the void volume of SEC experiments; and, by mass photometry, this species forms a monodisperse distribution of albeit larger, but still relatively small, molecules. Our mass photometry analysis supports the SEC, X-ray crystallographic and circular dichroism data demonstrating remodelling of ParDE1 complexes.

### Thermally driven ParE1 toxin release induces DNA cleavage

Despite our efforts, attempts to demonstrate ParE1 release during remodelling had so far failed, likely due to an inability to capture the small amounts available protein that are briefly in solution before precipitation occurs. Noting that the ParDE1 complex we initially isolated showed a higher than basal level of activity against gyrase (Figures 1A and B), and putting this in context with our biophysical data on remodelling, we concluded that the gyrase poisoning was likely a result of ParE1 toxin liberated by ParDE1 complex remodelling during the assay. To confirm and improve upon this result, we decided to repeat the biochemical analysis of ParDE1 in gyrase DNA relaxation assays, starting with a pure ParDE1 heterotetramer sample. Using the biochemical assay as a read-out would allow observation of the small amounts of soluble ParE1 released by remodelling, without concern for low ParE1 solubility at higher concentrations.

**Figure 7.**
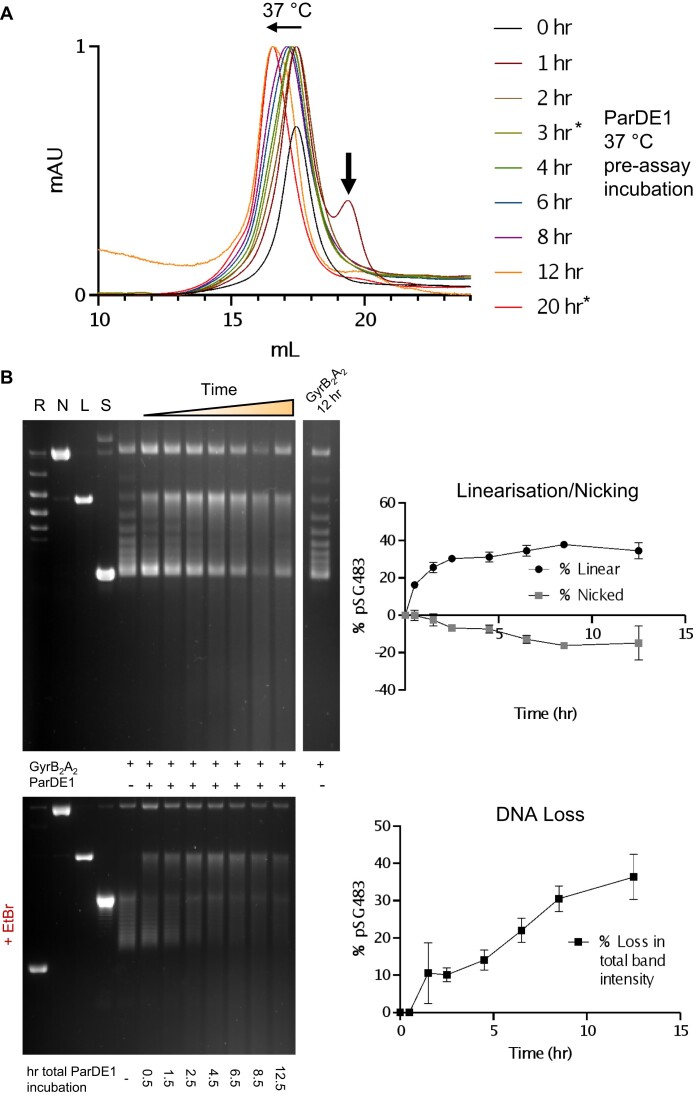
ParDE1 induced cleavage through thermally driven toxin liberation. (**A**) Analytical SEC traces for ParDE1 37°C incubation time course, starting with heterotetrameric complex (0 hr, solid black). A 100 μl sample of incubated ParDE1 (100 μM) was injected onto a Superose 6 10/300 GL column using an Åkta Pure system at each listed time point. Horizontal arrow indicates the general direction of peak shifting. Vertical arrow indicates the peak within the 1 hr SEC experiment that could represent free ParE1. * denotes SEC samples not used in the subsequent biochemical assay due to practicalities of the timings involved. (**B**) ParDE1 induced gyrase-dependent DNA cleavage assay. GyrB_2_A_2_ was reconstituted, diluted, and stored on ice. ParDE1 (10 μM final concentration) from each time point was added to constant GyrB_2_A_2_ (31.25 nM) and Supercoiled (S) plasmid DNA (12.5 nM). Presence (+) or omission (-) of GyrB_2_A_2_ and ParDE1 is detailed between the agarose gels for each lane. A 12 h GyrB_2_A_2_ only assay is included to ensure enzyme stability throughout the time-course. ParDE1 total incubation time at 37°C (pre-assay incubation time point + assay incubation) is shown below the gels (h). Control lanes represent Supercoiled (S), Linear (L), Nicked (S), and Relaxed (multiple topoisomers) (R) plasmid DNA. Assays are presented on 1.4% Agarose 1x TAE gels (run with ethidium bromide (+EtBr) as stated, or post-stained) alongside graphical analysis of percentage Linear/Nicked DNA and total percentage loss in band intensity per lane (+EtBr, obtained by densitometry) against time (hr). Incubation time for ParDE1 is quantified below the gels. Assays shown are representative of triplicate experiments, and data points and error bars represent the mean and SD of triplicate data.

A gyrase DNA cleavage assay was performed following pre-incubation of ParDE1 at 37°C to promote toxin release, and remodelling was concomitantly monitored by analytical SEC (Figure 7). ParDE1 analytical SEC was performed on the hour at the presented time points (Figure 7A). The starting, 0 hr, sample was a single peak positioned at the appropriate *V*_e_ (∼17.3 ml) for the heterotetrameric ParDE1 peak 2 complex, as expected (Figure 7A). Incubation at 37°C caused a gradual shift in the positioning towards peak 1 until the entire peak elutes at the *V*_e_ of the heterohexamer (∼16.1 ml) (Figure 7A). It is worth noting that the 3 hr, and final 20 h time-points were not tested in the corresponding biochemical assay due to practical time constraints during the assay, however, the bulk of the fraction is remodelled by 12 h (Figure 7A). Interestingly, at the 1 h time-point a clear peak was again fleetingly observed at the appropriate *V*_e_ (peak 3) for the ParE1 toxin (Figure 7A, thick black arrow).

At the indicated time-points, incubated ParDE1 was used to perform a gyrase DNA relaxation assay, to a final concentration of 10 μM ParDE1 as per the standard protocol for all presented relaxation assays (Figure 7B). A new relaxation reaction was set-up for each time-point using the pre-diluted stock of GyrB_2_A_2_. Due to protocol, the reaction itself provided an extra 30 minutes of incubation at 37°C, alongside a different buffer environment. The relaxation reaction is clearly inhibited at the 0-hr pre-incubation point with the notable presence of linear species DNA (Figure 7B, 0.5 h total incubation). This result is similar to that first previously observed (Figures 1A and B), but in this experiment there was an increased level of linearisation, perhaps due to using homogeneous heterotetrameric ParDE1 as starting material. The relaxation reaction becomes almost fully inhibited over the pre-incubation time-course as linearisation also increases, whilst the level of nicking decreases (Figure 7B). The area below the supercoiled band is presented to also demonstrate the increasing levels of non-specific DNA cleavage (evidenced by the smearing pattern within the lane) (Figure 7B, +/- EtBr). This demonstrates a clear reduction in the total band intensity at increased incubation times (Figure 7B). Together, we observe increasing linearisation and increasing non-specific DNA cleavage leading to reduction in distinct species over the time-course. This correlates with the movement of the chromatographic ‘peak 2’ from the right-hand side starting point for ParDE1 heterotetramer (Figure 7A, black trace), toward the final ‘peak 1′ (Figure 7A, red trace), representing remodelled heterohexamer. Importantly, these effects are independent of the increasing age of the initial gyrase stock that was used throughout the assays, as this stock was tested for relaxation activity in the absence of ParDE1 at the final 12-h time-point (Figure 7B). Gyrase remained stable and active throughout the experiment; thus, these results are solely due to the addition of ParDE1.

**Figure 8.**
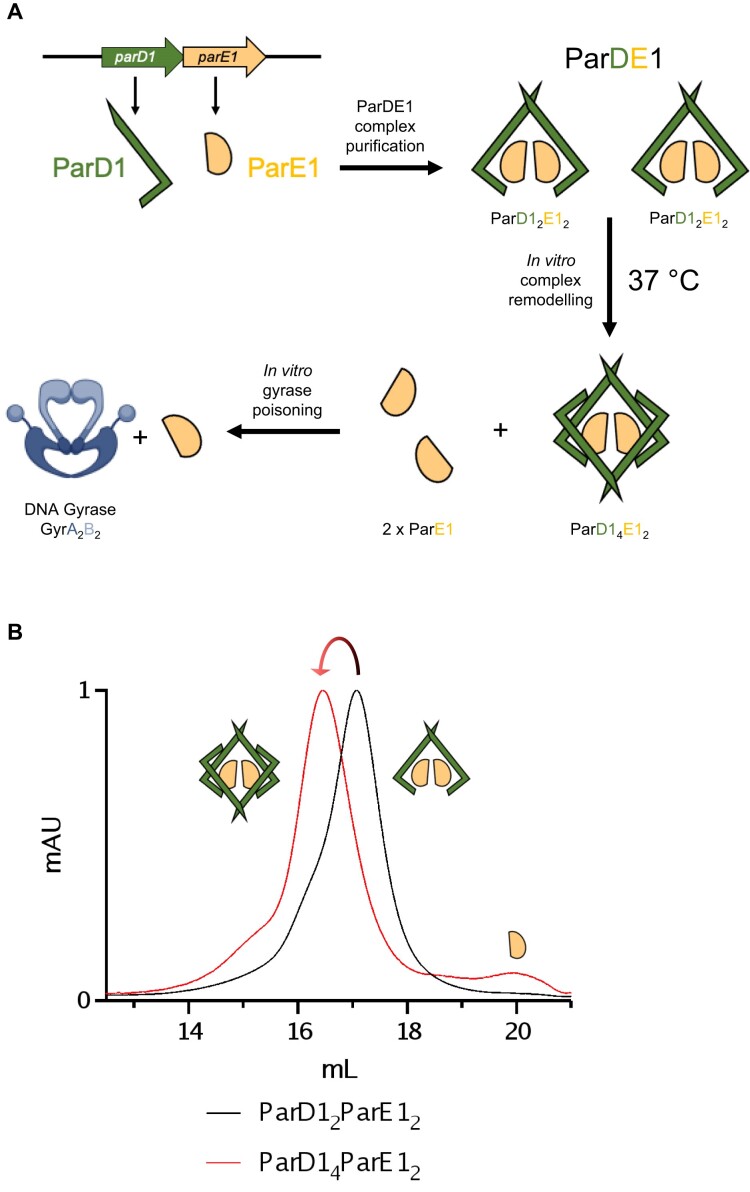
Schematic for *in vitro* ParE1 liberation through thermally driven ParDE1 complex remodelling. (**A**) Cartoon representation for the expression, initial complexing, and *in vitro* complex remodelling process hypothesised for ParDE1. ParD1 (green) and ParE1 (yellow) are expressed from a bicistronic operon and form a predicted ParDE1 heterotetrameric complex. Following purification, incubation at 37°C causes the entire fraction of ParD1_2_ParE1_2_ heterotetrameric complexes to remodel to form ParD1_4_ParE1_2_ heterohexameric complexes. Theoretically, two heterotetramers are required to generate a single heterohexamer, releasing two ParE1 toxin molecules. ParE1 toxin release was monitored through poisoning of gyrase. (**B**) Analytical SEC traces for the separated heterotetramer and heterohexamer fractions alongside their respective ParDE1 schematic. The heterohexamer trace is observed with a peak proposed to align with the size of the ParE1 toxin.

Based on these collected data we present a model for thermally driven *in vitro* conditional remodelling of ParDE1 complexes, leading to release of ParE1 toxins (Figure 8, Supplementary Figure S9). Remodelling allows conversion of two ParDE1 heterotetramer complexes into a single heterohexameric complex, and for every heterohexamer produced two ParE1 toxin molecules are liberated.

## Discussion

Our structures of both the ParDE2 complex (Figure 3) and ParDE1 complex (Figure 4) highlight key similarities and differences between the toxin and antitoxin protomers, alongside the quaternary complexes. The superfamily structure βααβββα (25) is largely present in both toxins. Sequence-based alignment of ParE2 and ParE1 returned an RMSD of 6.175 Å indicating low level sequence similarity, however, sequence-independent superposition returns a greatly improved RMSD value of 2.163 Å. This indicates that structure is more greatly conserved than sequence amongst ParE toxins. A notable difference between the toxin structures is evident at the C-termini (Figures 3 and 4). Unlike for ParE1, the C-terminal residues of ParE2 are predicted to form an α-helix (Figure 3D) and occupy the superfamily's conserved hydrophobic surface across the β-sheet core. This surface is instead occupied in the crystal by ParD2 (and corresponds to the site occupied by ParD1 in the ParDE1 structure) (Figures 3C and D). The importance of these C-terminal residues for toxicity of ParE2 has previously been demonstrated. Removing E95 – E105 (the C-terminal 10 amino acids), or making mutants E98A or R102A, renders the toxin ineffective (28). When considering the RelE/ParE superfamily (25), re-organisation of this helix to be positioned across the β-sheet core is also important in RelE toxins for the positioning of essential catalytic residues (61) and thus, ribonuclease activity when bound to the ribosome. ParE2 does not possess the canonical RelE catalytic core residues, therefore, the significance of the C-terminal helix requires further investigation.

The full ParDE2 complex structure is yet to be fully elucidated as the N-terminal dimerisation domain of ParD2 was cleaved during crystallisation (Supplementary Figure S4H). Our current model for the ParDE2 system is the ParD2_2_ParE2_2_ heterotetramer presented in Figure 3G. This model is supported by good alignment to the crystal structure (Figure 3G) and analytical SEC data (Supplementary Figure S4G). Interestingly, the heterotetramer model includes a ParD2 N-terminal dimerisation domain with structural similarity to the dimerisation domain of the *Lactococcus* phage TP901-1 Clear 1 repressor (PDB: 6FXA (69)), indicating DNA-binding capability. Interestingly, whilst we did not observe autoregulation for ParDE2 using the 1000 bp upstream, a previous report did show autoregulation, using only 363 bp of upstream sequence (28). Models for ParDE2 need to be developed further as this system is peculiar in its structure, especially in the ParD2 antitoxin and how it competes with the ParE2 C-terminal helix. Further to this, an open reading frame is identifiable upstream of the ParDE2 operon, the translated product of which shares 27% sequence similarity with ParD1 (though shorter at 51 amino acids vs ParD2 at 71 amino acids). It is plausible that this is the third member of a tripartite style ParDE system, similar to that seen for ParD2–PaaA2–ParE2 (70,71).

As for ParDE2, the structure of ParDE1 was also determined in an unexpected stoichiometry, forming a ParD1_4_ParE1_2_ heterohexamer (Figure 4). Analyses of the ParDE1 complex highlighted that the ParD1–ParE1 interaction is highly specific, not only interacting via the conserved superfamily interface as expected (Figure 4), but also via several tuned interactions in regions of lower sequence conservation (Supplementary Figure S7). ParD1 forms a dimeric CopG RHH motif through its N-terminal region (Figure 4). Investigating the structure of the CopG domain, of which there is one fully resolved in the structure, indicates a DNA-binding role as seen in the FitAB system (72), and suggests a likely model to explain the autoregulation we observed for ParDE1 (Figure 1C). Interestingly, CopG domains appear to permit interactions with elongated operators within promoter regions; the FitAB system employs two CopG domain that interact with operator sites ∼15 bp apart (PDB: 2BSQ (72)). In the *V. cholerae* ParDE system, three CopG domains exist back-to-back and create three DNA-binding sites for proposed enhanced DNA-binding through operator site interactions (PDB: 7R5A (73)). Further manual searches of the ParDE structures in the PDB indicated that ParDE complexes have increased plasticity in their stoichiometries; while 3KXE (27), 6X0A (74), and 6XRW (75) all exist as 2:2 heterotetramers, 5CEG (ParD_4_:ParE_4_ stoichiometry) (76) and 7R5A (ParD_6_:ParE_2_ stoichiometry) (73) exist as heterooctamers. Even more noteworthy is that the 7R5A structure resembles the ParDE1 complex with both fully and partially resolved ParD chains forming RHH CopG N-terminal dimers, however, an additional partially resolved ParD1 dimer places itself in-between the full-length ParD chains. Further to this, the 7B22 (73) structure of only the *V. cholerae* ParD chains forms a hetero-16mer (8 dimers) in a ring-like structure. Altogether, these results indicate that the ParD N-terminal domain permits higher-order stoichiometries to form. These related structures support our conclusions drawn from the observation of higher order complexes by SEC (Figure 6C) and mass photometry (Figure 6D).

The ParDE1 heterohexamer structure also supports our proposed model of *in vitro* ParDE1 complex remodelling for toxin release (Figure 8), based on the ParDE1 analytical SEC experiments, mass photometry, circular dichroism, and biochemistry (Figures 5–7). AlphaFold was used to successfully generate a heterotetramer model, which we predict to be the initial complex state for ParDE1 (Figure 4F). Interface analysis of the resulting heterohexamer complex seen in the crystal indicates that four interfaces are relevant in the formation of this higher order complex (Supplementary Figure S6). Through comparison to the search model (PDB: 3KXE (27)) and the ParDE1 heterotetramer AlphaFold prediction, alongside the PISA analysis, we suggest the heterohexamer structure be considered as a dimer of ParD1_2_ParE1 trimeric structures that interact mainly through their ParD1 CopG domains. This is supported by the structures of *V. cholerae* ParDE (PDB: 7R5A (73)) and *V. cholerae* ParD (PDB: 7B22 (73)) whereby the highly similar CopG domain multimerises in the same orientation seen for ParDE1. This observation has allowed us to develop our model for ParDE1 complex remodelling (Figure 8), to indicate likely swapping of ParE1 toxins (Supplementary Figure S9). We propose that during ParDE1 complex remodelling, the ParD1 CopG domain remains intact as a highly stable and conserved dimerisation domain. We propose that the CopG domains from two independent complexes interact through central ParD1 chains (blue and teal) (Supplementary Figure S9A and B). Displacement occurs at the relatively weak polar ParE1 – ParE1 interface (Supplementary Figure S6, interface iv), and due to steric clashes, the toxin pairs are reorganised. Why this process is driven by increasing temperature (Figures 6 and 7) is yet to be fully understood. Additionally, further work needs to be performed on elucidating the higher-order species of ParDE1 that evolves in the later stages of sample incubation (Figures 6C and 6D). At this moment, we do not know whether the ParDE2 complex would behave in a similar manner, remodelling in response to temperature. This is unlikely as purification and analysis via SEC routinely resulted in a single peak. It is more likely that ParDE2 exists in the typical 2:2 stoichiometry seen for ParDE and RelBE superfamily TA systems.

Though we have observed *in vitro* conditional remodelling of ParDE1 causing release of ParE1 toxin and inhibition of gyrase, it remains to be demonstrated whether this process has physiological relevance. This could be tested, for instance, by mutating residues at vital complex interfaces (Supplementary Figure S7) and performing *in vivo* analyses of both activation and toxicity. Current models for toxin activation rely either on antitoxin degradation by proteases, or increased transcription of the TA locus. If future work were to confirm *in vivo* application of conditional remodelling, this would suggest there should be room to consider alternate post-translational mechanisms of toxin release in cells. Notably, toxin release via degradation of the antitoxin has recently been disputed (77). Collectively, these findings advance our understanding of the type II TA complement of *M. tuberculosis*, examining systems that have been implicated in several roles contributing to the virulence and adaptation of *M. tuberculosis* during infection (30–31,78). ParE toxins remain of interest as potentially potent gyrase inhibitors worthy of further study.

## Supplementary Material

gkad1220_Supplemental_File

## Data Availability

The crystal structures of ParDE1 and ParDE2 have been deposited in the Protein Data Bank under accession numbers 8C24 and 8C26, respectively. All other data needed to evaluate the conclusions in the paper are present in the paper and/or Supplementary Data.
