# Supplementary material for: Toxin release by conditional remodelling of ParDE1 from Mycobacterium tuberculosis leads to gyrase inhibition
Source: Nucleic Acids Res. 2023 Dec 19;52(4):1909–29. doi: 10.1093/nar/gkad1220 (PMC10899793; doi:10.1093/nar/gkad1220)
Supplement: gkad1220_Supplemental_File [file gkad1220_supplemental_file.pdf]

SUPPLEMENTARY MATERIALS

Toxin release by conditional remodelling of ParDE1 from *Mycobacterium tuberculosis* leads to gyrase inhibition

Izaak N. Beck[a], Tom J. Arrowsmith[a], Matthew J. Grobbelaar[a], Elizabeth H. C. Bromley[b], Jon Marles-Wright[c], Tim R. Blower[a,*]

[a]Department of Biosciences, Durham University, Stockton Road, Durham, DH1 3LE, UK.

[b]Department of Physics, Durham University, Stockton Road, Durham, DH1 3LE, UK.

[c]Biosciences Institute, Newcastle University, Framlington Place, Newcastle upon Tyne, NE2 4HH

[*]To whom correspondence may be addressed. Email: timothy.blower@durham.ac.uk, tel: +44(0)1913343923.

Keywords: Toxin-antitoxin, topoisomerase, ParE, tuberculosis, protein dynamics

SUPPLEMENTARY FIGURES

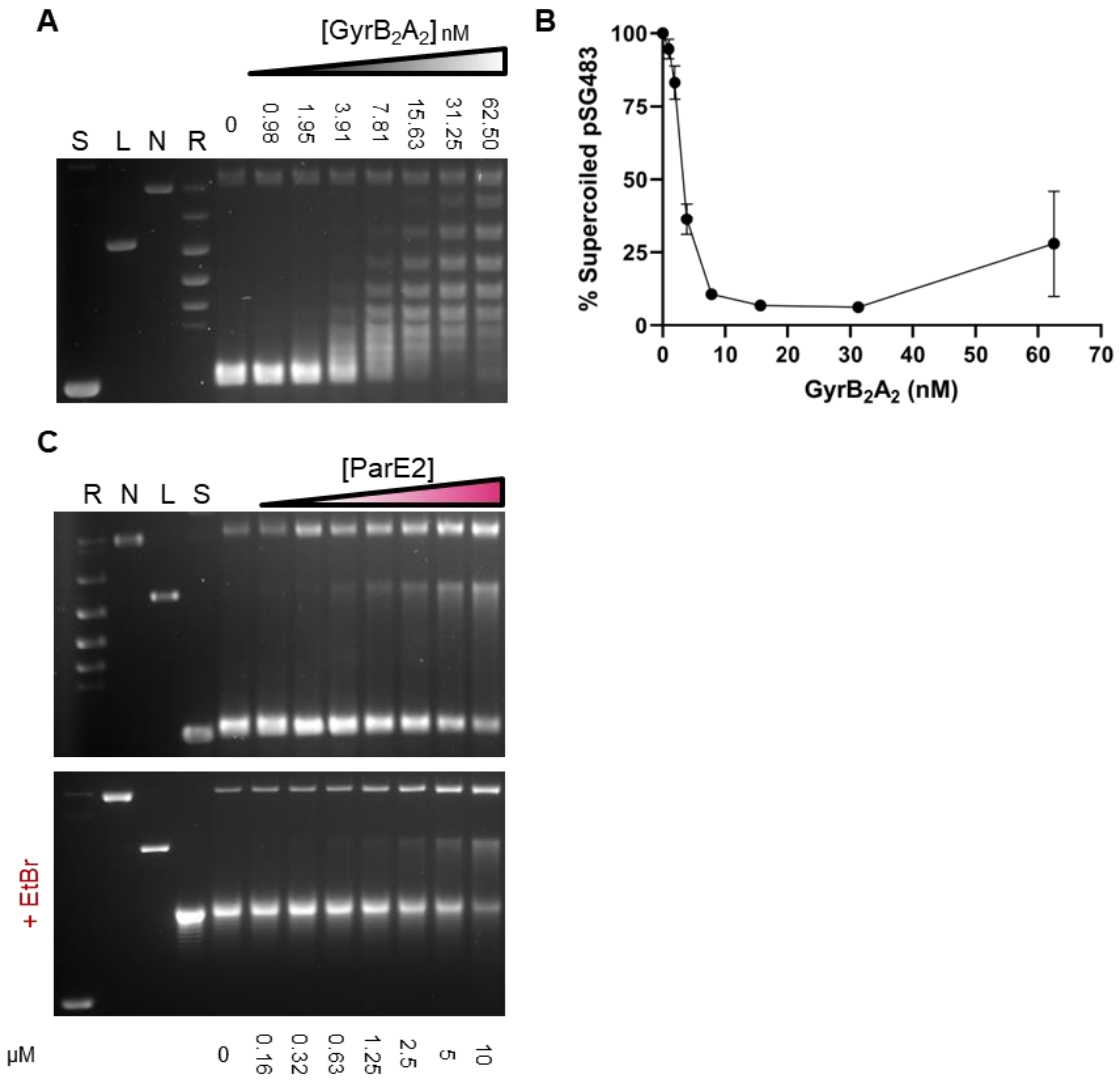

Supplementary Figure S1. *M. tuberculosis* gyrase DNA supercoil relaxation activity. (**A**) $GyrB_2A_2$ ATP-independent DNA relaxation reaction titration. (**B**) Removal of negative pSG483 supercoils by $GyrB_2A_2$ as shown in (**A**). (**C**) Analysis of background pSG483 linearisation by ParE2. Control lanes represent Supercoiled (S), Linear (L), Nicked (S), and Relaxed (multiple topoisomers) (R) plasmid DNA. Assays are presented on 1.4% agarose 1x TAE gel post-stained with EtBr or containing EtBr (+EtBr). The assays are representative of triplicate assays, and data points and error bars represent the mean and SD of triplicate data, respectively.

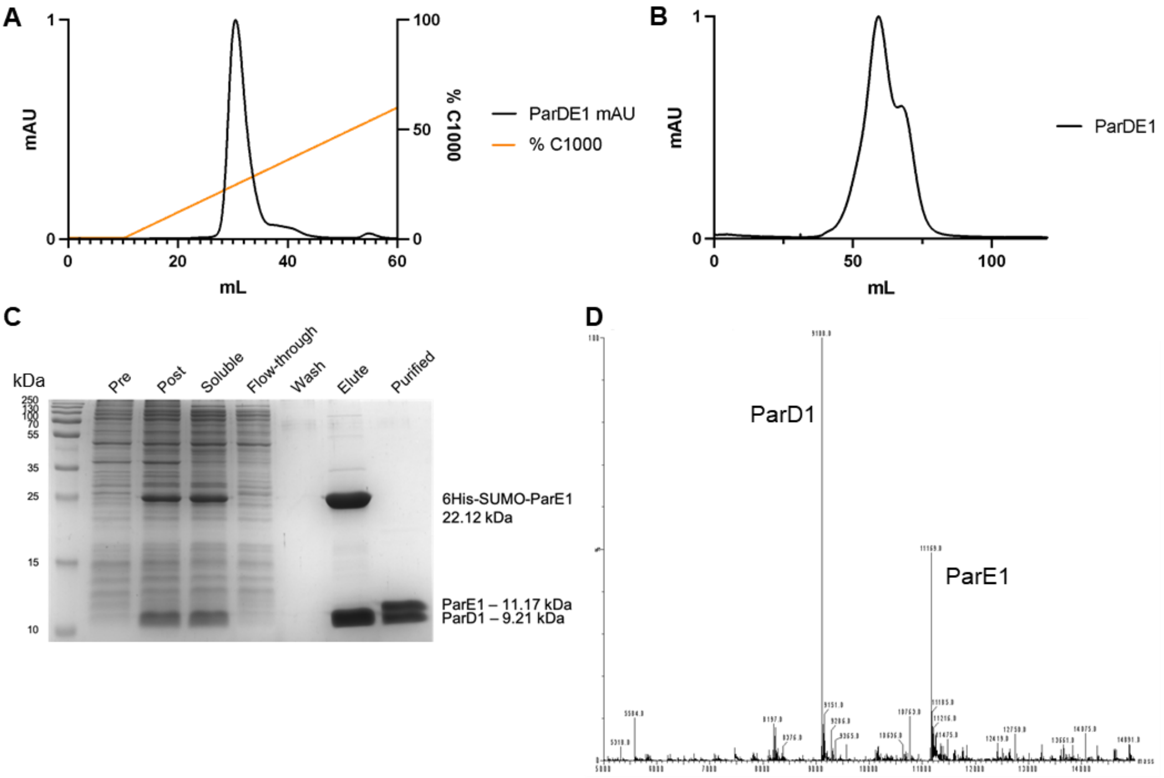

Supplementary Figure S2. ParDE1 complex production. (**A**) Anion exchange chromatogram from the purification of the ParDE1 complex. (**B**) SEC from the purification of the ParDE1 complex. (**C**) SDS-PAGE summary of the protein expression and purification procedure for the ParDE1 complex. Pre – whole cell lysate prior to protein expression induction; Post – whole cell lysate after protein expression induction showing the appearance of expression recombinant protein; Soluble – soluble protein fraction; Flow-through – protein fraction not bound to the initial Ni-NTA step; Wash – sample collected mid-way through the wash step from the initial Ni-NTA step; Elute – un-cleaved recombinant protein purity after initial Ni-NTA step; Purified – cleaved final product after anion exchange, tag cleavage and removal, and SEC. (**D**) ES^{+}-TOF Mass Spectrometry of the purified ParDE complex sample.

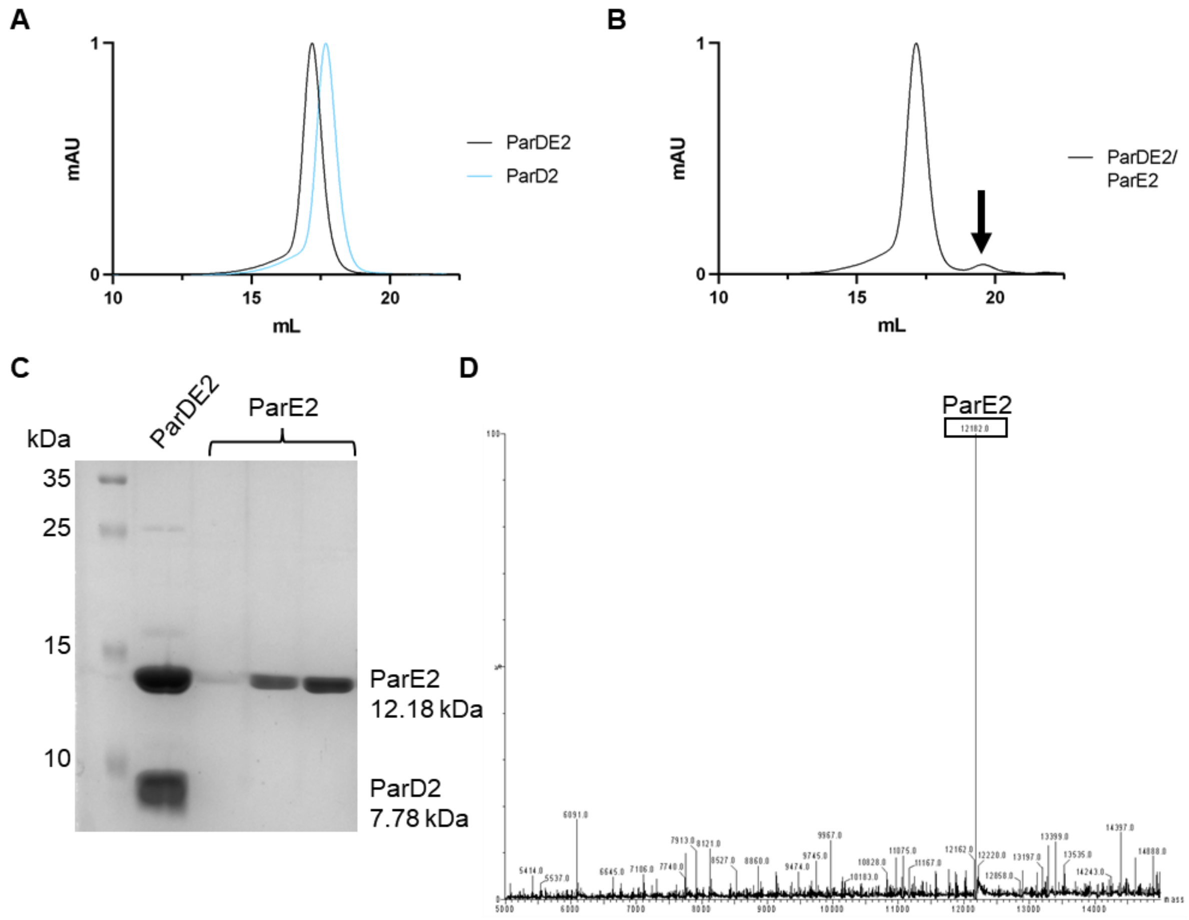

Supplementary Figure S3. ParE2 can be separated from ParDE2 complexes. (**A**) Analytical SEC traces for ParDE2 (black) and ParD2 (blue). (**B**) Analytical SEC trace of a ParDE2 purification containing free ParE2 toxin indicated by the vertical arrow. (**C**) SDS-PAGE analysis of purified ParDE2 and isolated ParE2 fractions from anion exchange that were subsequently pooled. (**D**) ES^+-TOF Mass Spectrometry analysis of the isolated ParE2 toxin.

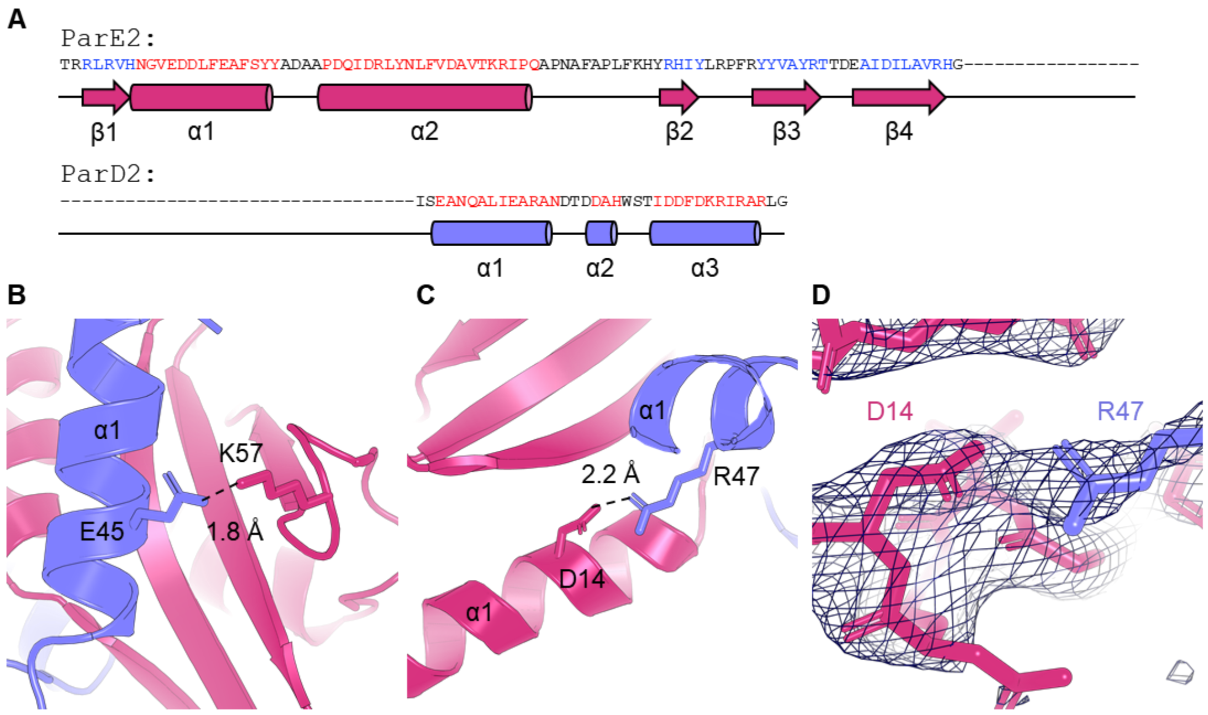

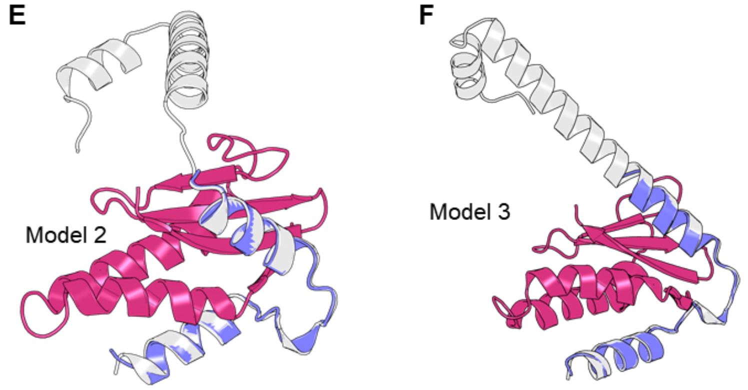

G

Sample	Calculated R_{st} (Å)	Observed R_{st} (Å)	Observed R_{st}/ Calculated R_{st}
ParDE2 (vs Model 2)	26.10[1]	30.27	1.16
ParDE2 (vs Model 3)	30.50[2]	30.27	1.05

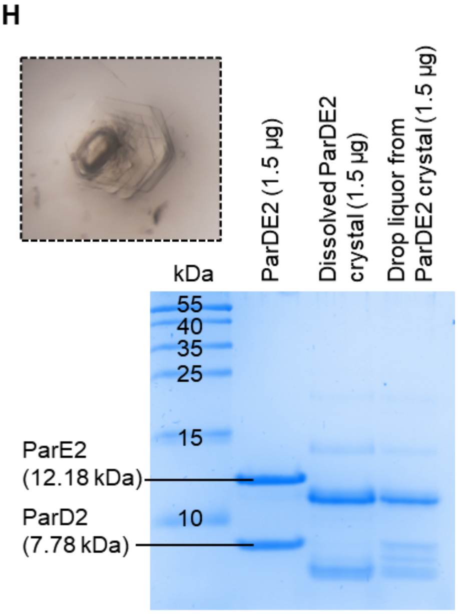

Supplementary Figure S4. Analysis of ParD2-ParE2 interactions. (**A**) Secondary structure schematics of ParE2 and ParD2. Secondary structure is displayed underneath the protein sequence, whereby amino acids constituting α helices are in red, and those constituting β strands are in blue. Secondary structure elements are coloured as per their crystal structure counterpart and represented by tubes for α helices and arrows for β strands and are labelled accordingly. Dashes represent unmodelled

regions. (**B** and (**C**) Ionic bonding interactions within the complex. (**D**) ParE2 D14 – ParD2 R47 interaction with F_o-F_c electron density map contouring at 2σ. (**E**) Enhanced view of ParD2 from Model 2 (Figure 2F) aligned to the ParDE2 crystal structure. (**F**) Enhanced view of ParD2 from Model 3 (Figure 2G) aligned to the ParDE2 crystal structure. Crystal structure colouring is as previously presented. AlphaFold ParD2 models are coloured light grey. (**G**) Table of ParDE2 protein Stokes Radius (R_{st}) calculations, observations, and comparisons. [1]R_{st} for AlphaFold Model 2 of $ParD2_2ParE2_2$ (heterotetramer); [2]R_{st} for AlphaFold Model 3 of $ParD2_2ParE2_2$ (heterotetramer). R_{st} values were generated using HullRad (Fleming and Fleming, 2018). Comparison of observed/calculated is coloured green if within 10% of the predicted ratio, yellow if > 10 ≥ 25%, and red if > 25%. (**H**) SDS-PAGE analysis of ParDE2 crystals compared to the input ParDE2 solution. Inset shows an image of the ParDE2 crystals.

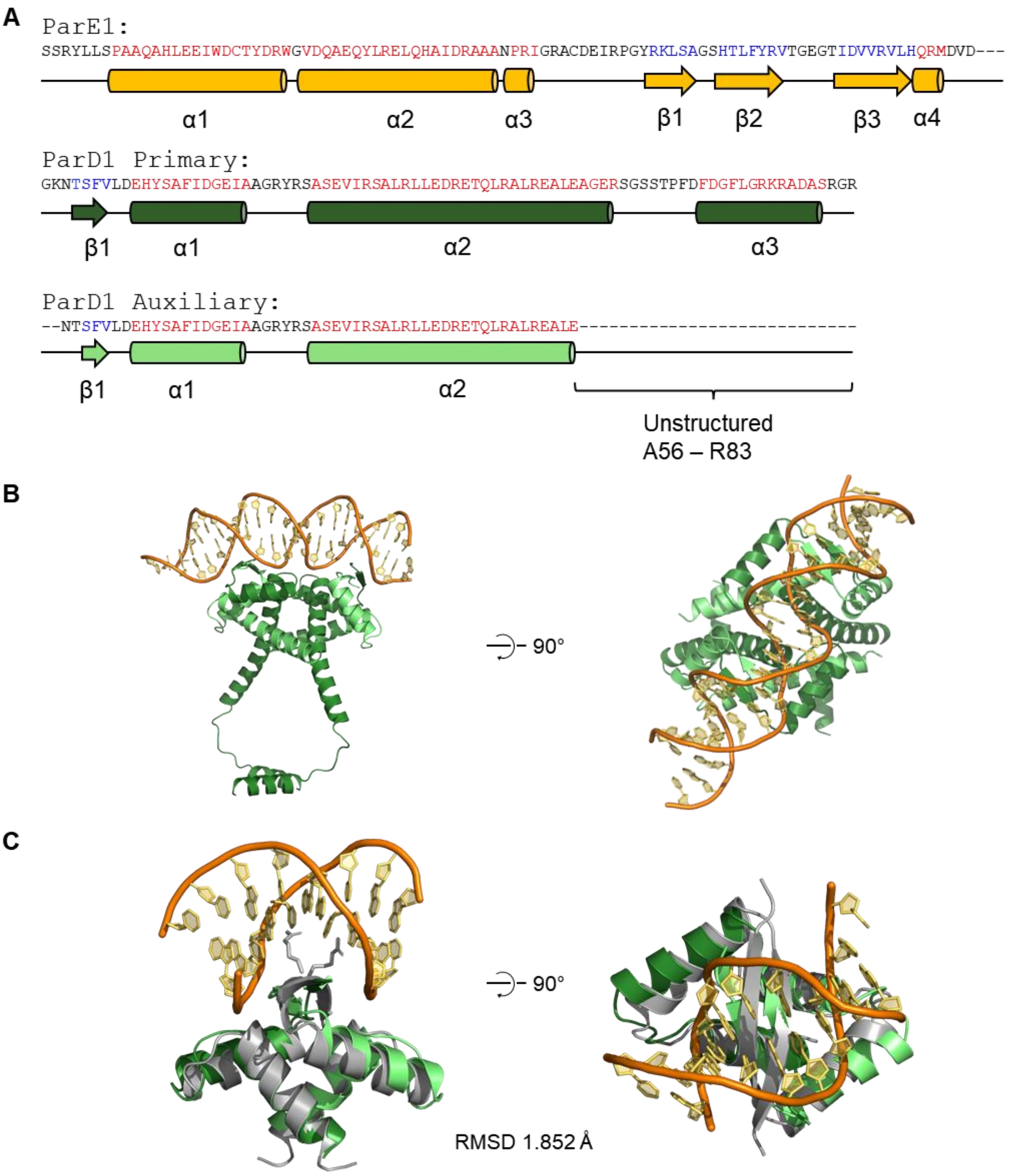

Supplementary Figure S5. Modelling ParD1-DNA. (**A**) Secondary structure schematics of ParE1, ParD1 primary, and ParD1 auxiliary proteins. Secondary structure is displayed underneath the protein sequence, whereby amino acids constituting α helices are coloured red, and those constituting β strands are coloured blue. Secondary structure elements are coloured as per their crystal structure counterpart and represented by tubes for α helices and arrows for β strands and are labelled accordingly. Dashes represent unmodelled regions. (**B**) DNA-bound ParD1 tetramer model (coloured as per Figure 4A) generated using PDB 1EA4. (**C**) DNA-bound model for a single RHH domain from ParDE1 generated using PDB 1B01, and then aligned back to PDB 1B01 (coloured grey).

A

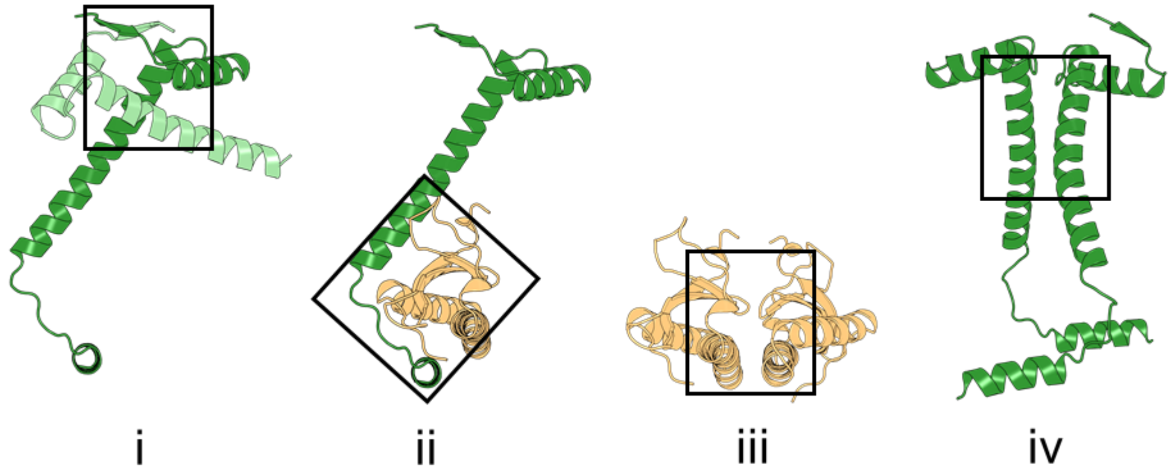

B

Interface	Interface area (Å²)	$\Delta^{i}G$ kcal/mol	$\Delta^{i}G$ P-value	N^{HB}	N^{SB}	N^{DS}	CSS
i	1560.4	-22.4	0.140	20	13	0	1.000
ii	1458.0	-12.5	0.243	27	19	0	1.000
iii	919.3	-1.4	0.481	12	6	0	0.079
iv	491.3	5.9	0.941	19	17	0	0.053

Supplementary Figure S6. Analysis of ParD1-ParE1 interactions. (**A**) Interfaces of the ParDE1 complex analysed by PISA. i: Primary ParD1 – Auxiliary ParD1 interface formed by an antiparallel β sheet; ii: Primary ParD1 – ParE1 interface; iii: ParE1 – ParE1 interface; iv: Primary ParD1 – Primary ParD1 interface; (**B**) PISA analysis of interfaces i – iv. Interface area is calculated in square angstroms ($Å^2$); $\Delta^{i}G$ – solvation energy on folding (free energy gain); N^{HB} – Number of hydrogen bonds within the respective interface; N^{SB} – Number of salt bridges within the respective interface; N^{DS} – Number of disulphide bonds formed within the respective interface; CSS – Complex Significance Score ranges between 0 – 1 as interface relevance to complex formation increases.

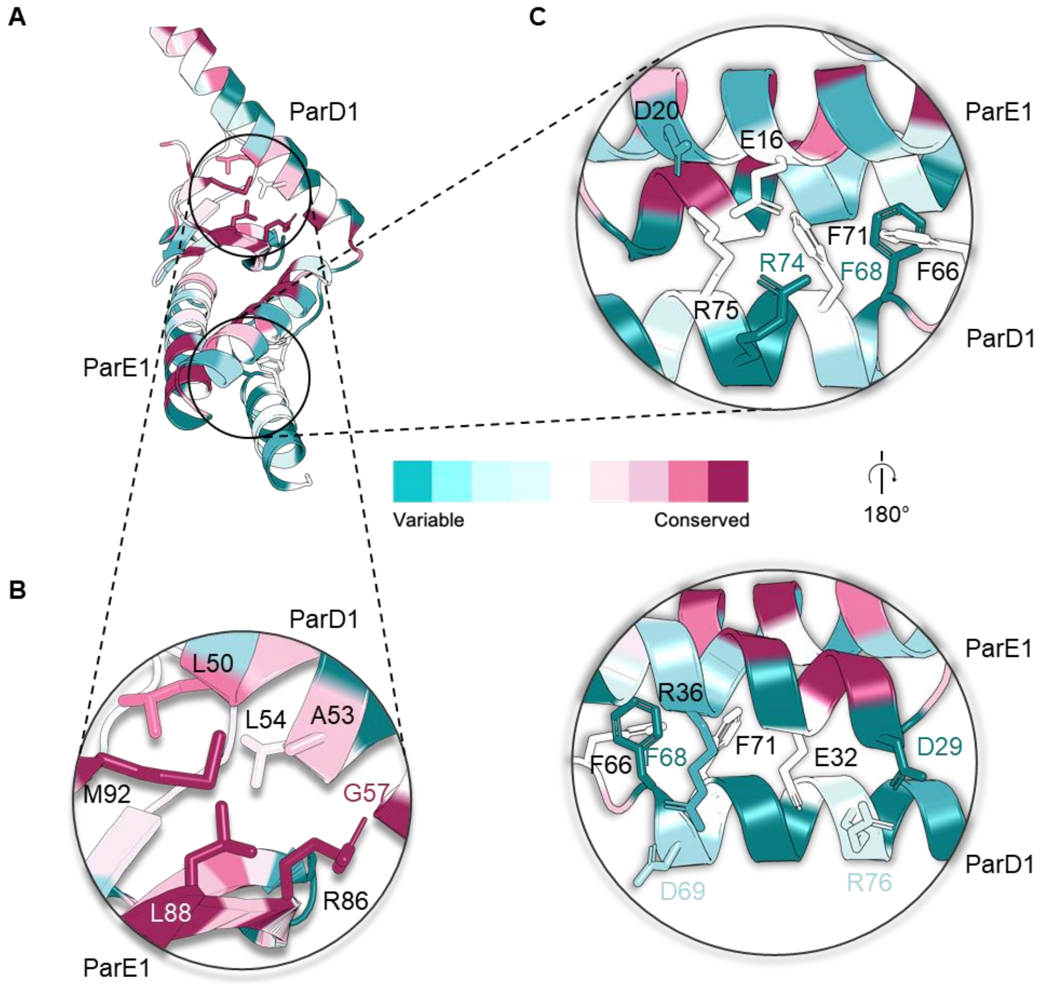

D

ParD1	ParE1	Å
A53	L88	3.6
A53	M92	4.0
L50	L88	4.1
L50	M92	4.5
G57	R86	4.5
L54	L88	4.8

E

ParD1	ParE1	Å
D69	R36	3.2
R74	E16	3.5
K75	E16	2.7
K75	D20	2.4
R76	E32	3.9
R76	D29	3.0

Supplementary Figure S7. Conserved residues within ParDE1 interactions. (**A**) Cartoon ParDE1 dimer with ConSurf colouring, highlighting interactions between ParD1 and ParE1 chains by conservation. ConSurf colouring scheme (shown as a gradient) represents amino acid conservation scores. (**B**) Expanded view of the top black circle from (**A**) highlighting conserved amino acid interactions. (**C**) Expanded view of the lower black circle in (**A**) with additional 180° rotated view highlighting tuned, lower conservation, amino acid side chain interactions. (**D**) List of side chain interactions and distances from (**B**). (**E**) List of side chain interactions and distances from (**C**).

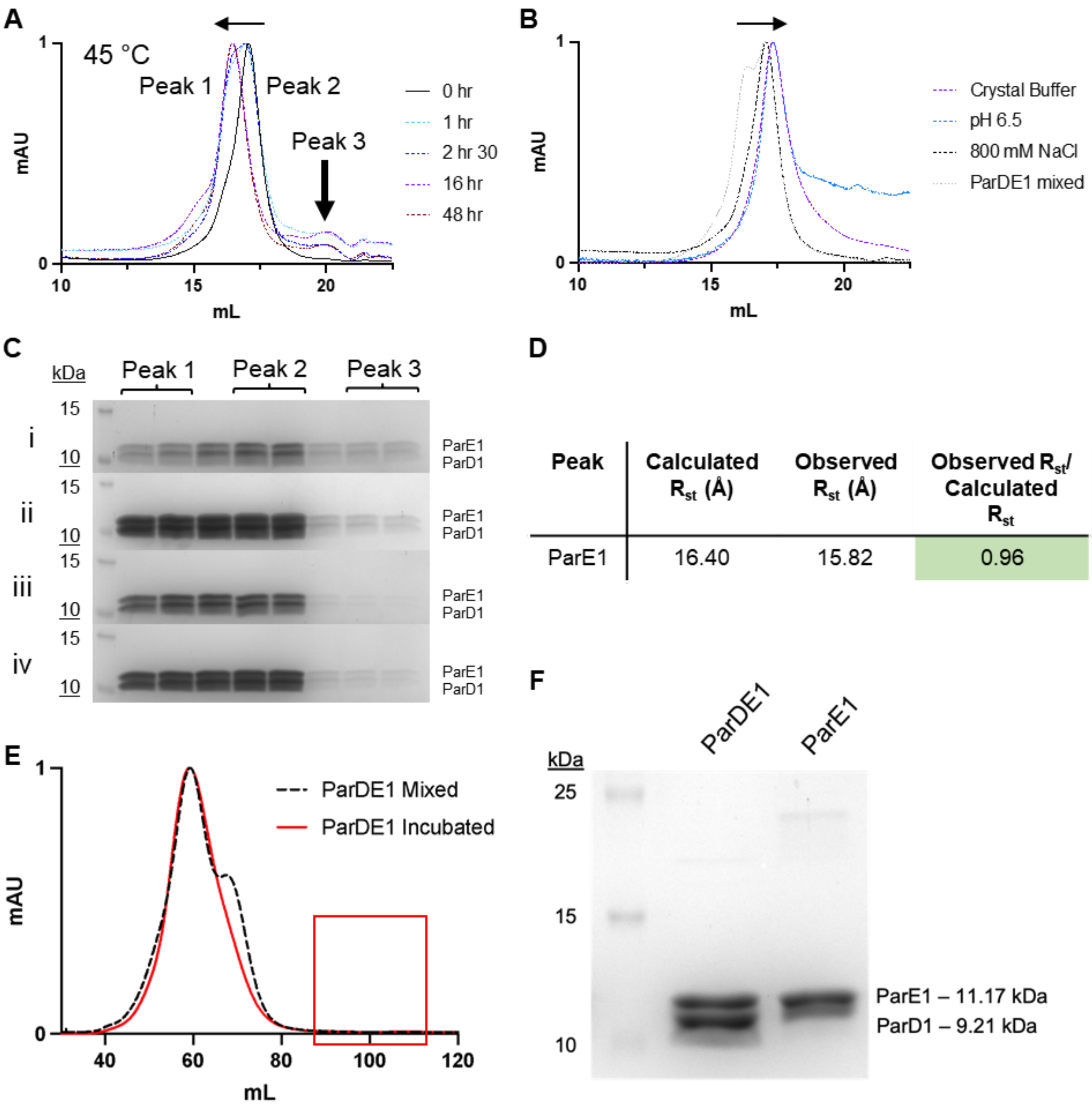

Supplementary Figure S8. ParDE1 undergoes conditional complex remodelling and potentially releases ParE1. Analytical SEC traces for ParDE1 (**A**) 45 °C incubation alongside the starting, 0 hr trace (solid black). Vertical arrow indicates potential free ParE1 “Peak 3”. (**B**) Buffer changes, presented alongside the mixed sample from Figure 6A (light grey). Horizontal black arrows indicate general direction of peak shifting, vertical arrows indicate the generation of the third peak, predicted to be liberated ParE1 toxin. (**C**) SDS-PAGE analysis of fractions aligning to observed peak 1 – heterohexamer; peak 2 – heterotetramer; peak 3 – ParE1: i – 0 hr from Figure 6A; ii – 15 mg from Figure 6B; iii – 37 °C 16 hr from Figure 6C; iv – 45 °C 16 hr from **A**. (**D**) Table for ParE1 Stokes Radius (R_{st}) calculations, observations, and comparisons. Calculated R_{st} for ParE1 was generated using HullRad (Fleming and Fleming, 2018) and the structure solution available in the AlphaFold database (Varadi *et al.*, 2022).

Comparison of observed/calculated is coloured green if within 10% of the predicted ratio, yellow if > 10 ≥ 25%, and red if > 25%. Chromatograms are representative of single repeats and are normalised between 0 – 1 for presentation and comparison. Graphs are cropped to the appropriate scale (10 – 22.5 mL). (**E**) Attempt for large-scale purification of ParE1; sephacryl S-200 HR SEC trace of the soluble fraction from the incubation of 10 mg/mL ParDE1 complex at 37 °C for 16 hr (solid red) alongside the mixed peak purification example (dashed black) (Supplementary Figure S2B). Red box indicates the expected range for the ParE1 toxin peak. (**F**) SDS-PAGE of the purified ParDE1 complex from SEC presented in A (ParDE1 lane) alongside the precipitated fraction from overnight incubation of ParDE1 at 37 °C (ParE1 lane).

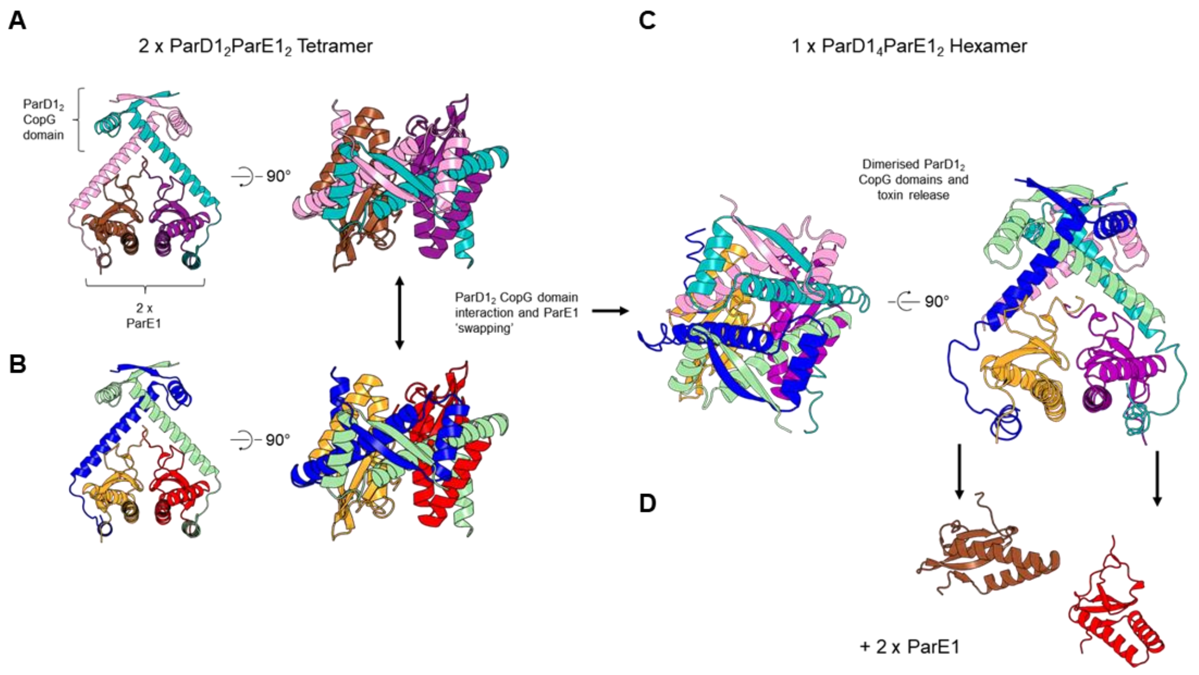

Supplementary Figure S9. Structural model for ParDE1 complex remodelling and ParE1 toxin release. (**A**) AlphaFold generated ParD1$_2$ParE1$_2$ heterotetramer with ParD chains coloured teal and pink and ParE chains coloured brown and purple. The ParD1 dimer CopG domain is labelled. (**B**) AlphaFold generated ParD1$_2$ParE1$_2$ heterotetramer with ParD chains coloured light green and blue and ParE chains coloured light orange and red. (**C**) AlphaFold generated ParD1$_4$ParE1$_2$ heterohexamer after complex remodelling has occurred through dimerisation of the tetramer CopG domains and re-pairing of ParE1 toxins (now purple and light orange, from **A** and **B**, respectively). Internal facing ParD1 chains (teal and blue, from **A** and **B**, respectively) remain fully structured in complex with toxins. (**D**) Liberated brown and red ParE1 toxins, from **A** and **B**, respectively. Toxins are released, resulting in the transition to disordered/unstructured ParD1 C-termini for external pink and light green ParD1 chains (from **A** and **B**, respectively).

Supplementary Tables

Supplementary Table S1. Plasmids used in this study.

Plasmid	Notes	Reference
pTRB316	6His-TEV-*gyrB* (*rv0005*), Km	(3)
pTRB696	6His-TEV-*gyrA* (*rv0006*), Km	(3)
pTRB568	*parD2* (*rv2142A*), Ap	Genscript, (this study)
pTRB569	*parD1* (*rv1960c*), 6His-SUMO-*parE1* (*rv1959c*), Ap	Genscript, (this study)
pTRB570	*parD2* (*rv2142A*), 6His-SUMO-*parE2* (*rv2142c*), Ap	Genscript, (this study)
pJEM15	Promoterless LacZ, Km	(36)
pJEM15-$P_{rv1960c/rv1959c}$	Promoterless LacZ with 1000 bp upstream of ParDE1 cloned in, Km	Genscript, (this study)
pJEM15-$P_{rv2142A/rv2142c}$	Promoterless LacZ with 1000 bp upstream of ParDE2 cloned in, Km	Genscript, (this study)
pGMC	Anhydrotetracycline (ATc) inducible vector, Sm	(35)
pGMC-*parD1*	*parD1* (*rv1960c*), Sm	Genscript, (this study)
pGMC-*parE1*	*parE1* (*rv1959c*), Sm	Genscript, (this study)
pGMC-*parDE1*	*parDE1* (*rv1960c, rv1959c*), Sm	Genscript, (this study)
pGMC-*parD2*	*parD2* (*rv2142A*), Sm	Genscript, (this study)
pGMC-*parE2*	*parE2* (*rv2142c*), Sm	Genscript, (this study)
pGMC-*parDE2*	*parDE2* (*rv2142A, rv2142c*), Sm	Genscript, (this study)
